# Abnormal T-Cell activation and cytotoxic T-Cell frequency discriminate symptom severity in myalgic encephalomyelitis/chronic fatigue syndrome

**DOI:** 10.1186/s12967-025-07507-x

**Published:** 2025-12-10

**Authors:** Ji-Sook Lee, Eliana Lacerda, Caroline Kingdon, Ella Abken, Giada Susannini, Hazel M. Dockrell, Luis Nacul, Jacqueline M. Cliff

**Affiliations:** 1 Department of Infection Biology, London School of Hygiene & Tropical Medicine, London, UK; 2 Clinical Research Department, London School of Hygiene & Tropical Medicine, London, UK; 3 Centre for Inflammation Research and Translational Medicine, Department of Biosciences, Brunel University of London, Uxbridge, UB8 3PH UK

**Keywords:** ME/CFS, Symptom severity, CD8^+^ T cells, Mucosal-associated invariant T cells, Cytotoxicity, T cell activation

## Abstract

**Background:**

Myalgic encephalomyelitis/chronic fatigue syndrome (ME/CFS) is a debilitating but poorly-understood disease. ME/CFS symptoms include immune system effects alongside incapacitating fatigue and post-exertional disease exacerbation. Symptom severity can range from mild to severe and whilst symptoms can fluctuate, few people fully recover.

**Methods:**

Immunological profiles of people living with ME/CFS were analysed by flow cytometry, focusing on cytotoxic cells, to determine whether people with mild/moderate (*n* = 43) or severe ME/CFS (*n* = 53) expressed different immunological markers. Flow cytometry data were tested for normality and the two clinical groups were compared by t-test or Mann-Whitney U-test as appropriate.

**Results:**

People with mild/moderate ME/CFS had increased expression of cytotoxic effector molecules alongside enhanced proportions of early-immunosenescence cells, determined by the CD28^-^CD57^-^ phenotype, indicative of persistent viral infection. In contrast, people with severe ME/CFS had higher proportions of activated circulating lymphocytes, determined by CD69^+^ and CD38^+^ expression, and expressed more pro-inflammatory cytokines, including interferon-γ, tumour necrosis factor and interleukin-17, following stimulation in vitro, indicative of prolonged non-specific inflammation. These changes were consistent across different cell types including CD8^+^ T cells, mucosal associated invariant T cells and Natural Killer cells, indicating generalised altered cytotoxic responses across the innate and adaptive immune system.

**Conclusions:**

These immunological differences likely reflect different disease pathogenesis mechanisms occurring in the two clinical groups, opening up opportunities for the development of prognostic markers and stratified treatments.

**Supplementary information:**

The online version contains supplementary material available at 10.1186/s12967-025-07507-x.

## Background

Myalgic encephalomyelitis/chronic fatigue syndrome (ME/CFS) is a debilitating disease with a prevalence of 0.1 to 2.2% in Europe [1]. It is characterized by profound fatigue and post-exertional malaise (PEM), along with sleep, neurocognition, immune and autonomic nervous system dysfunctions which significantly impact on quality of daily life [2, 3]. ME/CFS affects all age groups, including children and adolescents, and is more common in women than men [4]. The symptom pattern is heterogeneous between individuals and can change within individuals through time [5], the majority of people living with ME/CFS (PWME) do not fully recover [6, 7]. People with mild/moderate ME/CFS can often engage in some daily activities albeit with limitations, but those who are severely affected are usually housebound, and in some cases bedbound [8]. Disease time-course patterns are diverse: more than half the PWME experience fluctuating (relapsing/remitting) symptom severity, particularly those with more mild/moderate disease; some have a gradual improvement in symptoms while others deteriorate long-term. As the pathophysiological mechanisms of this disease are still to be determined, diagnosis of ME/CFS is still based on clinical features [9], and the degree of disease severity has been classified by Carruthers et al. into mild, moderate, severe, and very severe groups [10]. The aetiology of ME/CFS is not fully understood; its onset often follows acute infections, but we know little about the triggering events for symptom fluctuation and the regulatory events which enable remission. We also do not know whether mild, moderate and severe ME/CFS are caused by the same disease mechanisms, or whether different symptom manifestations represent different underlying causal events. Better understanding of these differences would enable stratification of PWME, the development of personalised treatments and the possibility of elusive diagnostic biomarkers.

Immune system abnormalities have been investigated in PWME, to attempt to understand the disease aetiology. Altered T cell phenotype and activation, and impaired natural killer (NK) cell cytotoxicity have been reported [11–15], suggesting an increased susceptibility to infections, including reactivation or persistency of human herpesviruses [16, 17], or gut microbiome dysbiosis. However, recent reports showed no association or inconsistent results of the immune cell phenotype and function [18–20]: most studies have analysed small cohorts, often with diverse clinical presentations, and clear identification of immune alterations in ME/CFS remains elusive. Overlapping symptom and laboratory results have been described between people with long COVID following SARS-CoV-2 infection and PWME, suggesting mechanistic similarity between the conditions [21, 22].

Clinical and immunological parameters have been shown to be associated with disease severity in ME/CFS. In clinical analyses, we previously reported handgrip strength as a potential diagnostic biomarker for ME/CFS [23], while blood creatine phosphokinase concentration was able to discriminate between people with mild/moderate and severe ME/CFS [24]. Immunological studies have sought to determine serum cytokine signatures in ME/CFS associated with disease severity and disease duration. Montoya et al. reported 13 inflammatory cytokines levels in serum were correlated with severity of disease [25], while people with recent onset of ME/CFS had prominent elevation of serum proinflammatory and anti-inflammatory cytokines which were not present in people with longer-term ME/CFS [26]. Recently, natural regulatory autoantibodies were reported to be associated with symptom severity in people with ME/CFS which was triggered by infection [27]. Further, an abnormal increase of intracellular giant lipid organelles in peripheral immune cells has been reported in people severely affected with ME/CFS [28]. From these studies it can be inferred that investigation of immunological parameters which distinguish between people with mild/moderate (ME-MM) or severe (ME-SA) ME/CFS will help our understanding of ME/CFS pathomechanisms. In our previous study, in a large cohort of ~ 250 well-characterised PWME, we found alterations in the phenotype of CD8^+^ T cells, with an increase in effector memory cell and decrease in terminally differentiated cell proportions, alongside an elevated frequency of mucosal associated invariant T cells (MAITs) and of CD8^+^ MAITs, in people severely affected with ME/CFS:^18^ this reported study did not include a full analysis of cytotoxic cells, nor their functional capacity to produce cytokines or cytotoxic mediators such as perforin or granzymes, nor how the leukocyte subsets related to each other, nor whether the differences observed between clinical groups remain stable through time, potentially indicating different disease pathogenesis mechanisms.

Here, we aimed to determine how immunological parameters, particularly MAIT and CD8^+^ T cell phenotype and function, were associated with clinical symptom scores in PWME, to identify prognostic biomarkers and to facilitate better understanding of complex disease pathogenesis. To achieve this, we analysed samples from a cohort of people with ME-MM or ME-SA which were collected longitudinally (2 to 5 times at approximately 6-month intervals), and compared the flow cytometry data generated with eleven clinical parameters which were adapted for assessment of disease severity in PWME.

## Methods

### Study approval

This study was conducted according to the principles of the Declaration of Helsinki. Study approval was obtained from the London School of Hygiene & Tropical Medicine (LSHTM) Research Ethics Committee 16 January 2012 (Ref.6123) and the National Research Ethics Service (NRES) London: Bloomsbury Research Ethics Committee 22 December 2011 (REC ref.11/10/1760, IRAS ID: 77,765). Written informed consent was obtained from all study participants prior to inclusion in the study.

### Participant enrolment

A total of 96 participants were enrolled in the study. All participants with ME/CFS had a formal diagnosis and met either the Canadian Consensus Criteria [9] or CDC-1994 criteria [29]; many fulfilled both [30]. Exclusion criteria were: taken antiviral medication or drugs known to alter immune function or any vaccinations in the preceding 3 months; a history of acute or chronic infectious diseases such as hepatitis B or C, tuberculosis, HIV (but not herpes virus or other retrovirus infection); another chronic disease such as cancer, coronary heart disease or uncontrolled diabetes; a severe mood disorder; being pregnant or breastfeeding in the preceding 12 months; morbid obesity (BMI ≥ 40). Symptom questionnaires and clinical appointments were used to assess the severity of disease, with participants characterised as having mild/moderate ME/CFS (ME-MM) or being severely affected (ME-SA) [8]. Severely affected study participants were visited at home by the clinical team. The study was designed as a longitudinal study, with blood samples collected five times with at least six month gaps between time points. However, the COVID-19 pandemic negatively affected both recruitment and follow-up, so participants were variously sampled 2, 3, 4 or 5 times. Both female and male participants were included in the study. ME/CFS predominantly affects women, and our study cohort of 96 individuals included 76 women (79%) and 20 men (21%) which reflects this difference in susceptibility. The participants’ demographic information is shown in Table 1. The sample size was based on our previous report [18]. Venous blood from participants was collected into heparinized vacutainers, and transferred to the UCL/RFH Biobank at the Royal Free Hospital London for isolation and cryopreservation of peripheral blood mononuclear cells (PBMCs).

**Table 1:** Demographic characteristics of the study population at baseline

Variables		Mild/Moderate (n = 43)	Severe (n = 53)	*P* value
Sex	Male	8	12	0.235
	Female	35	41	
Age, yr (mean)	Male	45.1	42.8	0.287
	Female	36.2	39.5	
BMI, Kg/m^2^ (mean)	Male	28.2	22.0	0.001
	Female	25.2	20.1	
Handgrip strength (mean)	Male	30.8	29.6	0.115
	Female	19.7	15.5	
PCS (mean)	Male	26.0	20.2	< 0.0001
	Female	31.4	19.2	
MCS (mean)	Male	44.0	42.5	0.039
	Female	42.0	45.8	

Note: BMI – Body Mass Index, PCS – Physical Component Summary (from SF-36 v^2^_TM_), MCS – Mental Component Summary (from SF-36 v^2^_TM_). * *P*-value was calculated for the comparison between the mild/moderate group and the severely affected group

### Flow cytometry

Cryopreserved PBMCs were transferred to LSHTM for analysis, where they were stored in liquid nitrogen until use. Samples from different time-points from one individual were processed together, whereas participants’ samples were randomly selected for processing in batches. For processing, PBMC vials were defrosted in a 37 °C water bath for 1 min, then the contents were transferred to 12 ml warm RPMI 1640 including 10% foetal bovine serum (RPMI/FBS) and centrifuged at 650 g for 15 mins. After 2 washes with RPMI/FBS, cells were rested at 37°C/5% CO_2_ for 30 mins. Cells were then pelleted by centrifugation and re-suspended in RPMI/FBS, after which cells were counted and viability ascertained using the Countess3 automated cell counter (Invitrogen, MA, USA). One million cells were added to wells of a 96 well V-bottom plate for “ex vivo” staining, where extra- and intra-cellular staining processes with target antibodies were performed: antibodies used, including Research Resource Identifiers, are shown in Supplementary Table S1. Another one million cells/sample were incubated overnight in RPMI/FBS at 37°C/5% CO_2_ in preparation for “In vitro stimulation” the next day. A total of six staining panels were used for flow cytometric analysis of each sample, including five ex vivo and one in vitro panel (Supplementary Table S2).

For extracellular staining, cell suspensions were centrifuged at 750 g for 5 mins, supernatants were removed by flicking, and cell pellets were loosened by gentle vortex. Cells were washed with FACS buffer containing bovine calf serum and sodium azide (Cell Staining Buffer, BioLegend, CA, USA) and centrifuged. Cells were first incubated with 1:200 prediluted MR1 tetramer in 20 μl FACS buffer (loaded with 5-OP-RU and 6-FP as a negative control) obtained from the NIH Tetramer Core Facility [31], for 40 mins at room temperature in the dark. Cells were then washed and stained with the appropriate cocktail of antibodies and live/dead stain in FACS buffer at 4 °C for 20 min: these antibody staining panels included anti-human CD3/CD4/CD8/CD56/CD161/TCR Vα7.2 for T cell, MAIT and NK cell phenotyping throughout, along with “activation/exhaustion” and “memory/differentiation” specific marker staining panels (Supplementary Table S2). After incubation, 200 μl of FACS buffer was added, and cells pelleted by centrifugation.

For the “function” panel, for both ex vivo and in vitro stimulated cells, following the extracellular staining 75 μl of cell fixation and permeabilization solution (BD biosciences, NJ, USA) was added to each well, and incubated for 15 mins at room temperature in the dark. Then, using Perm/Wash buffer (BD biosciences), cells were washed and stained with anti-cytokine/cytotoxic granule antibodies for 30 mins at room temperature in the dark. Similarly, for the “Transcription factor” panel, cells were fixed and permeabilized using the FOXP3 Transcription factor staining buffer set (eBioscience, CA, USA) for 30 mins at room temperature in the dark, then stained with anti-human transcription factor antibodies for 1hour at room temperature in the dark, in permeabilization buffer. After intracellular or intranuclear staining, cells were centrifuged at 930 g for 5 mins, then suspended with FACS buffer, and analysed by flow cytometry the following day.

For the in vitro stimulation, following the overnight incubation, “cell stimulation cocktail” (phorbol 12-myristate 13-acetate (PMA) and ionomycin, eBioscience) was added to the cells in the 24-well plate. After 1hour, protein transport inhibitor cocktail (brefeldin A and monensin, eBioscience) was added to each well, then incubated for 4 hours more. After incubation, the supernatant was removed by pipetting, and all cells were transferred to a 96 well V-bottom plate, and stained as described above.

To validate the multiparametric flow cytometry panels, they were first optimised on samples from healthy control donors. To monitor the consistency of flow cytometry across different batches of experiments, we included PBMCs from healthy controls in all experiments. FMOs (fluorescence minus one) and isotype controls were included for the gating strategy in all experiments.

An LSRII cytometer (BD) was used for cell data acquisition. We aimed to acquire 2x10^5^ CD3^+^ T cells in the LSRII to facilitate analysis of the MAIT cells which are a minor cell population: only populations containing ≥ 20 events are reported due to technical limitation. Flow cytometry data analysis was done using FlowJo software version 10.8.1 (Tree star Inc. Ashland, USA).

### Cytomegalovirus IgG measurement

Serological assay of anti-CMV IgG in plasma was performed using a quantitative ELISA kit (Demeditec Diagnostics (Kiel, Germany) following the manufacturer’s protocol. The antibody concentration (Unit, U) was calculated using sample observance value and cut-off value, as instructed in the protocol. For a qualitative evaluation of IgG antibody to CMV, the concentration was interpreted as follows: concentration ≤ 9 U as a negative, concentration ≥ 11 U as a positive, and concentration between 9 U and 11 U as equivocal.

### Statistics

Presented values (cell frequency or Median Fluorescence Intensity (MFI)) were calculated as the mean value of repeated measures from those acquired at different timepoints (either 2,3,4,5 measures/participant). Datasets were tested for normality using the Shapiro-Wilk test. For two group comparisons, the unpaired t-test or Mann-Whitney U-test were used for normal or non-normal distribution data respectively. For paired sample comparisons, the paired t-test for parametric data or Wilcoxon matched-pairs test for non-parametric data were used. Multiple testing corrections were not applied due to the interdependent nature of the variables quantified. Spearman correlation tests were conducted to determine the association between two variables. *p* < 0.05 was considered statistically significant, and tests were 2-sided. P-values are shown as exact numeric value, except when *p* < 0.001. Gender differences between clinical groups were analysed by Chi-squared test. All flow cytometry data analyses were performed using GraphPad Prism version 10.0.2.

## Results

### Study participants

The study population consisted of ninety-six individuals with ME/CFS, who were blood donors for the UK ME/CFS Biobank [30] (Table 1). All participants had a medically confirmed ME/CFS diagnosis from the UK National Health Service, and were rigorously assessed by the study clinicians to assure compliance with the Centers for Disease Control (CDC-94) [29] and/or Canadian Consensus Criteria [9]. Participants were extensively clinically assessed, including measurement of creatinine phosphokinase in blood and handgrip strength reading by dynamometer [23]. Clinical scores were calculated for each of the following seven domains based on the Canadian Consensus Criteria: post-exertional malaise, sleep dysfunction, pain, neurological/cognitive dysfunction, autonomic dysfunction, neurocognitive dysfunction and immune system manifestations. Forty-three (44.8%) of those research participants were classified at recruitment as having mild/moderate symptoms and were ambulatory (ME-MM), whereas 53 (55.2%) had severe symptoms and were house- or bed-bound (ME-SA).

Repeated blood samples were taken from each participant, to remove day-to-day fluctuations in cell number and function, and the average of these taken as the data point in the flow cytometric analysis. Participants were reassessed at each study visit: while there was some degree of fluctuation in disease severity at different timepoints, particularly within the ME-MM group, this was not of a sufficient degree to lead to changes in severity classification.

### Mucosal-associated invariant T cell frequency is elevated in ME-SA

**Fig. 1:**
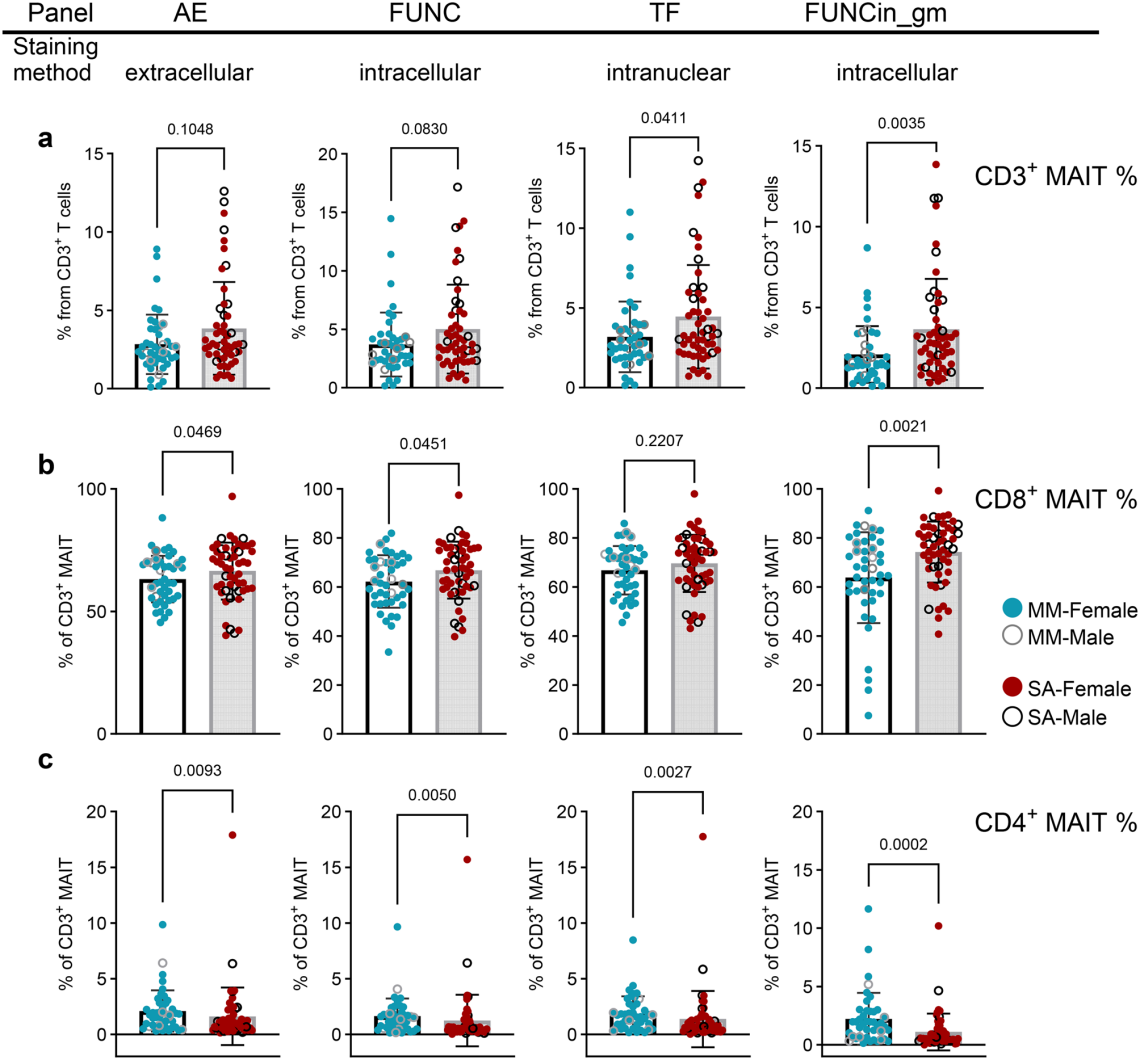
Differential frequencies of mucosal-associated invariant T cell subsets in mild/moderate (*n* = 43) and severe ME/CFS (*n* = 53). PBMC from people with mild/moderate (mm) or severe (SA) ME/CFS symptoms were analysed by flow cytometry, to determine the proportions of (**a**) total MAIT cells within the CD3^+^ T cell compartment, (**b**) CD8^+^ T cells within the CD3^+^ MAITs and (**c**) CD4^+^ T cells within the CD3^+^ MAIT compartment. PBMC samples were analysed by four separate flow cytometry staining panels: in common, PBMCs were stained extracellularly for immune cell profiles, then specific stain methods were engaged. “ae”: Activation/Exhaustion markers; “func”: functional markers; “TF”: transcription factor markers; “FUNCin_gm”: functional marker staining following incubation overnight in culture medium. Each dot represents the average value across all the samples collected at different time points for one individual study participant. Mean values and sd were plotted in graphs. The data for CD8^+^MAIT (% in ‘FUNC’, ‘TF’ in figure 1b) cells were normally distributed and analysed by an unpaired t-test; all other datasets were not normal and were analysed by the mann-whitney test. Statistical significance was set at *p* < 0.05

*C*ryopreserved peripheral blood mononuclear cells (PBMCs) were thawed and analysed by flow cytometry: the gating strategy is shown in Supplementary Figure S1. Initially, we sought to confirm whether PWME had altered frequencies of T cell subsets and NK cells, depending on the severity of their symptoms, in this robust repeated-measures analysis. Firstly, we compared the frequencies of T cells (identified as CD3^+^) and T cell subsets (CD4^+^ T cells, CD8^+^ T cells, CD4CD8 Double Negative (DN) T cells, CD4^+^CD8^+^ Double Positive (DP) T cells, CD3^+^CD56^+^ NKT-like cells), as well as NK cells (identified as CD3^-^CD56^+^) and CD8^+^CD56^+^NK cells (“NK8 cells”) in ex vivo PBMCs from people with ME-MM or ME-SA, and found there were no differences between the two clinical groups (Supplementary Figure S2a-2 h). In a previous study [18], we found that the frequency of circulating total MAIT cells (phenotype: CD3^+^TCR Vα7.2^+^CD161^++^) was elevated in ME-SA, compared to healthy controls, multiple sclerosis and ME-MM, and also that the frequency of CD8^+^ MAITs within total MAITs was significantly elevated in ME-SA compared to ME-MM and healthy controls. In this study, we included fluorescently labelled MHC related protein-1 (MR-1) tetramer staining for better identification of the MAIT subset within the CD3^+^ T cell population [31]. As shown in Fig. 1, we confirmed the trend that the ME-SA group had elevated frequencies of CD3^+^ MAIT cells and of CD8^+^ MAITs, which is a major subset of total MAITs, compared to those in the ME-MM group. These patterns were reproducible through four different flow cytometry staining panels utilised, although statistical significance varied by staining methods such that *p* < 0.05 in three of four panels in each case: the differences became more apparent after overnight resting in culture medium as part of the “in vitro stimulation” methodology. Interestingly, the frequency of CD4^+^ MAIT cells was significantly higher in the ME-MM group (Fig. 1c) in all the flow cytometry staining panels. We also confirmed that the frequencies of NK cells and the minor DN T cell subsets were reproducible across the different staining methods, including extracellular-only and additional intracellular staining: there was a slight increase in frequencies detected with the ‘function’ staining panel, but the pattern between the two clinical groups was highly similar across all staining methods (Supplementary Figure S2i). Interestingly, we observed significantly diminished expression of CD8, based on MFI, on CD8^+^ T cells in the ME-MM group compared with the ME-SA group (Supplementary Figure S3b). In some samples, a distinct CD8^intermediate^ cell population could be observed. We analysed the CD8^upper^ population and the CD8^intermediate^ populations separately, to check whether the frequency or CD8 expression level in those populations could underlie the decreased CD8 expression level overall in ME-MM group. The frequency and CD8 expression in the CD8^intermediate^ population were significantly higher in ME-MM, while those in the CD8^upper^ population were significantly higher in ME-SA (Supplementary Figure S3c-d).

### T cell and MAIT memory subsets show signs of early senescence in ME-MM

**Fig. 2:**
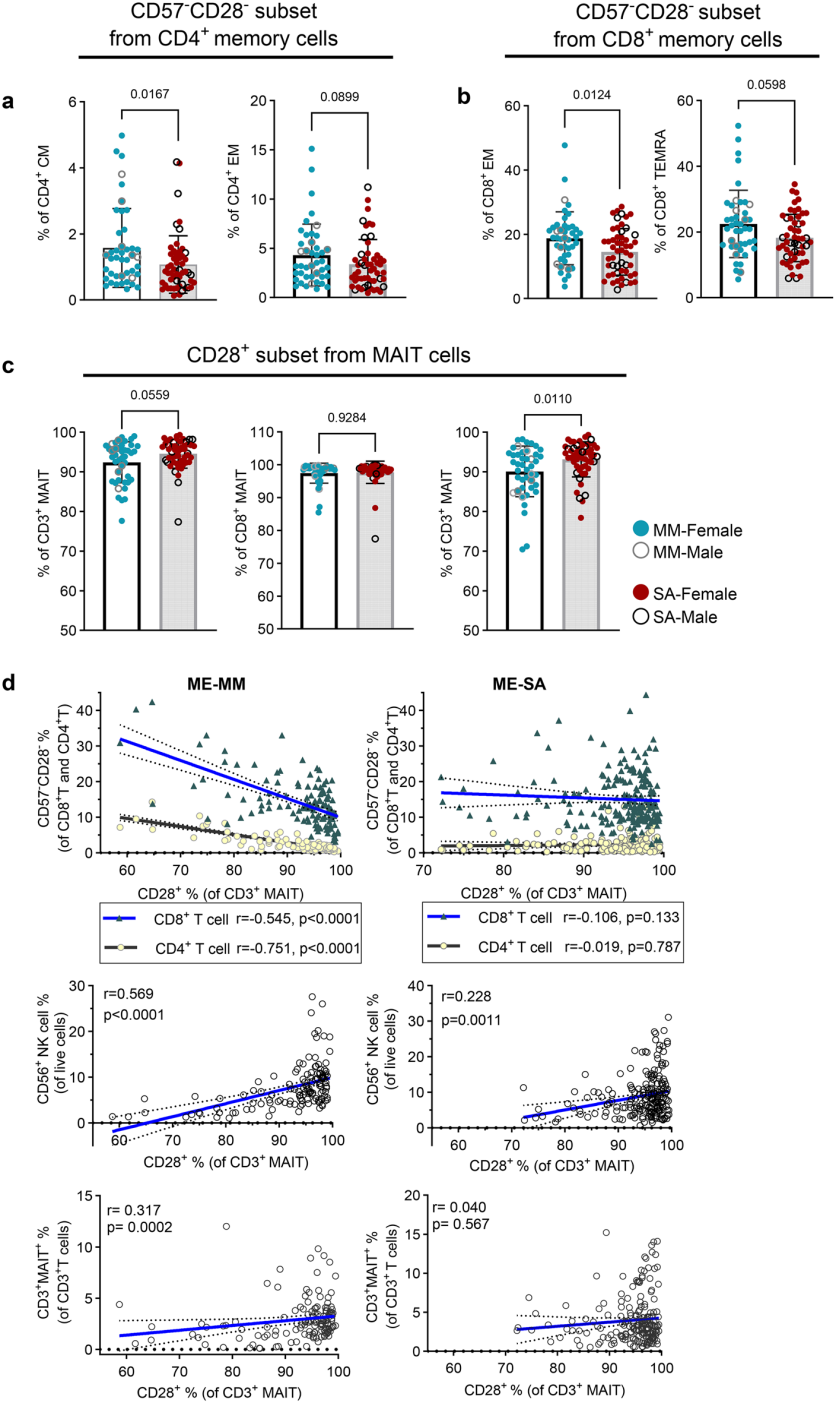
Differential expression of CD57^-^CD28^-^ subsets in memory T cells and CD28^+^ MAIT cells in mild/moderate (*n* = 43) and severe ME/CFS (*n* = 53). PBMC samples were analysed using the ‘memory/differentiation panel’ comprising CD45RA, CCR7, CD57, and CD28. (**a**) CD57^-^CD28^-^subsets (%) in central memory (CM) CD4^+^T cells (left) and effector memory (em) CD4^+^T cells (right). (**b**) CD57^-^CD28^-^ subsets (%) in em CD8^+^T cells (left) and terminally differentiated (TEMRA) CD8^+^T cells (right). (**c**) CD28^+^ subset (%) in total CD3^+^MAIT cells (left), in CD8^+^ MAIT cells (middle) and CD4^-^CD8^-^ MAIT cells (right). Each dot represents the average value across all the samples collected at different time points for one individual. Mean values and standard deviation (SD) are shown. All datasets were not in normal distribution and were analysed by the mann-whitney test. (**d**) correlation analysis of frequencies of CD28^+^ MAIT cells and CD57^-^CD28^-^ subsets in CD4^+^T cells and CD8^+^T cells (upper), NK cells (middle), and CD3^+^ MAIT cells (bottom). A total 335 samples (*n* = 132 for mm, *n* = 203 for SA) were used for the Spearman correlation test; the dashed line represents the 95% confident interval

We next investigated whether there were differences in the memory and differentiation states of T cells and MAITs from people within the ME-MM and ME-SA groups, by measuring naïve and memory cell markers (CD45RA, CCR7) and differentiation markers (CD28, CD57). Naïve cells were defined as CCR7^+^CD45RA^+^, CCR7^+^CD45RA^-^ as central memory (CM), CCR7^-^CD45RA^-^ as effector memory (EM) and CCR7^-^CD45RA^+^ as terminally re-expressing CD45RA effector cells (TEMRA). There were no significant differences in frequencies of naïve/memory subsets in CD4^+^ or CD8^+^ T cells between the ME-MM and ME-SA groups, nor were there any differences in the frequencies of CD57^-^CD28^+^, CD57^-^CD28^-^, CD57^+^CD28^-^ or CD57^+^CD28^+^ subsets (Supplementary Figure S4). Next, we analysed each CD4^+^ and CD8^+^T cell naïve/memory subset by CD57 and CD28 expression. In CD4^+^ T cells, > 95% of the naïve cells were CD57^-^CD28^+^, and the frequency of this phenotype reduced through the Naïve, CM, EM then TEMRA cell compartments, whereas the frequency of the CD57^+^CD28^-^ subset, which associated with senescence [32–34], gradually increased from the Naïve cells through CM then EM to TEMRA, although there were no significant differences between two clinical groups (Supplementary Figure S5). However, the frequency of the CD57CD28^-^ subset, which is considered a marker of early senescence alongside highly differentiated phenotype [33], was significantly higher in the CD4^+^ CM T cell subset in the ME-MM group compared to the ME-SA group, with a non-significant trend (*p* = 0.0899) towards higher frequency also in the CD4^+^ EM T cells in the ME-MM group (Fig. 2a). Moreover, a similar pattern was also seen in CD8^+^ T cells, with the frequency of CD57^-^CD28^-^ cells in the CD8^+^ EM and CD8^+^ TEMRA T cell subsets being significantly elevated in ME-MM compared to ME-SA (*p* = 0.012 in CD8^+^ EM, *p* = 0.0598 in CD8^+^ TEMRA) (Fig. 2b). CD3^+^ MAIT cells reportedly display an effector memory cell phenotype [35, 36]. Here, we found MAIT cells expressed CD28, but not CD57 on their surface. Interestingly, the frequency of CD28^+^ MAIT cells was reduced in ME-MM with a non-significant trend (*p* = 0.0559) towards higher frequency, compared to the ME-SA group (Fig. 2c left). The majority of CD8^+^ MAITs expressed CD28, without no significant difference between the two clinical groups; however, CD4^-^CD8^-^ MAIT cells had reduced frequency of CD28 expression in the ME-MM group compared to those from people with ME-SA (*p* = 0.010) (Fig. 2c right).

We then performed correlation analyses which showed differences between the ME-MM and ME-SA groups in the association between the frequency of CD28^+^ MAIT cells and of T cell CD57^-^CD28^-^ subsets: there was a negative correlation in the ME-MM group for both CD4^+^ (*r* = −0.751, *p* < 0.0001) and CD8^+^ (*r* = −0.545, *p* < 0.0001) CD57^-^CD28^-^ T cells and CD28^+^ MAITs (Fig. 2d). These observations were not observed in people with ME-SA. As MAITs cells act as innate-like T cells in response to microbial infection, we performed a correlation analysis between the frequency of NK cells, another innate immune cell type, and MAITs to determine whether these innate immune cells’ appearance together would affect the first line of defence in microbial infection, and found that the frequency of CD28^+^ MAITs was moderately correlated with the frequency of NK cells (*r* = 0.569, *p* < 0.0001) in ME-MM. Overall, this may indicate that reduced frequency of CD28 expression in MAITs from ME-MM is associated with higher frequency of CD57^-^CD28 T cell subsets and lower frequency of NK cells.

### Granzyme and/or perforin expressing CD8^+^ T cells and MAITs are reduced in people with ME-SA, ex vivo and following stimulation

**Fig. 3:**
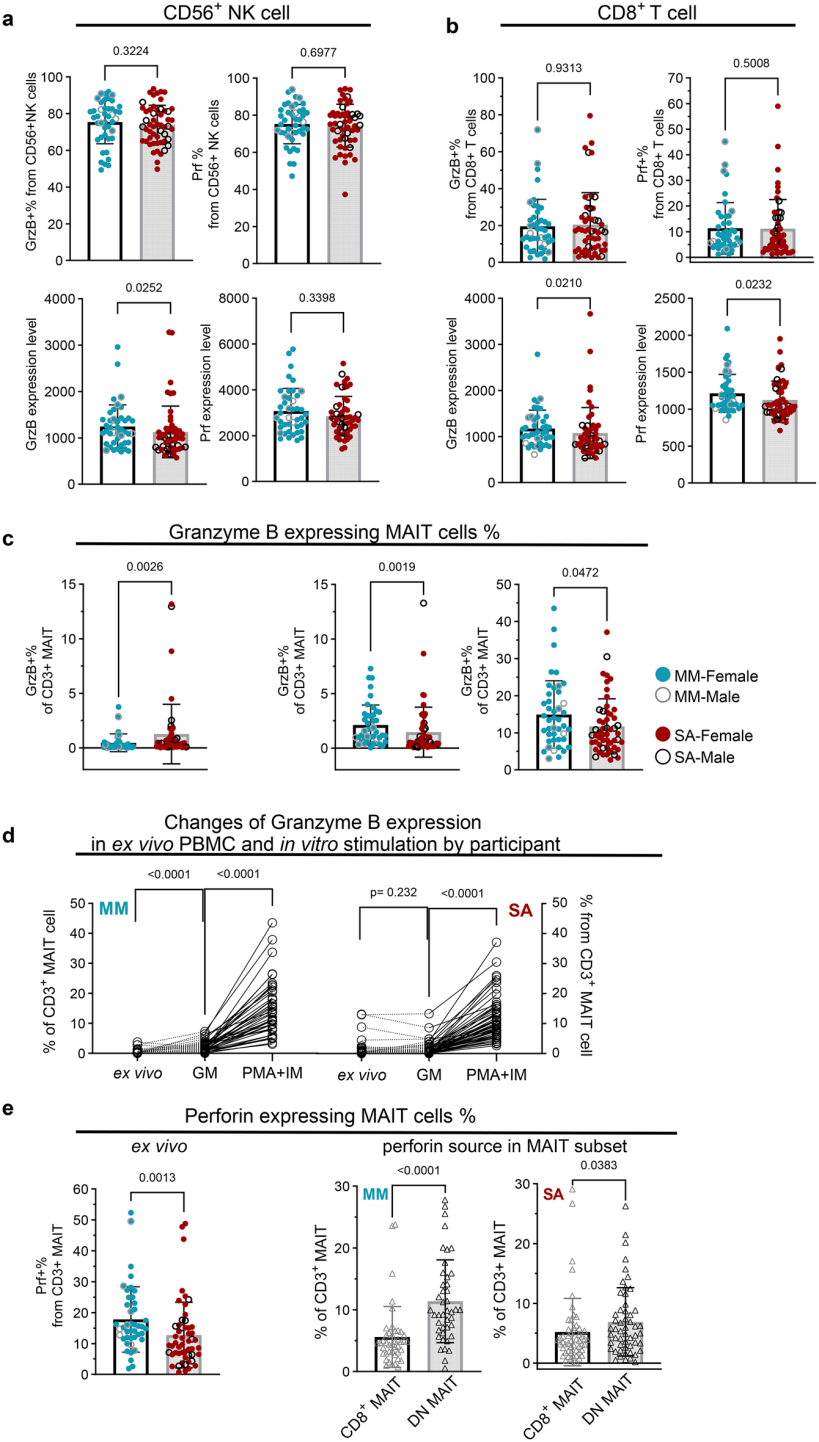
Ex vivo and in vitro analysis of expression of cytotoxic mediators in cytotoxic cells (NK cells, CD8^+^T cells and MAITs) from people with mild/moderate (*n* = 43) and severe ME/CFS (*n* = 53). Thawed PBMC samples were stained for intracellular granzyme B (GrzB) and perforin (prf). The cytotoxic molecule-expressing cell population was analysed by frequency (upper) and median fluorescence intensity (MFI, lower) of GrzB (left) and prf (right) in CD56^+^ NK cells (**a**) and CD8^+^T cells (**b**). (**c**) The frequencies of GrzB-expressing CD3^+^ MAITs were compared between ME-MM and ME-SA in ex vivo PBMC directly after thawing (left), resting overnight in culture medium (middle) and following 5 hours of stimulation with PMA and ionomycin after incubation overnight (right). (**d**) The data used in (**c**) were reanalysed by paired sample comparison (ex vivo vs GM or GM vs PMA+IM). (**e**) Prf expression in CD3^+^MAITs was compared between ME-MM and ME-SA groups in thawed PBMC (left), and the main source of the prf expression in CD3+MAIT cells was analysed in CD8^+^ MAITs cells and CD4^-^CD8^-^ DN MAITs in the ME-MM (middle) and ME-SA (right) groups. Each dot represents the average value across all the samples collected at different time points for one individual. Mean values and sd are shown. The data for prf (^+^%_CD56^+^NK) (panel 3a right) were normally distributed and analysed by an unpaired t-test; all other datasets were not normal and were therefore analysed by the Mann-Whitney test. The dataset with *p* < 0.05 was deemed significant. For the paired sample comparison in (**d**), the Wilcoxon matched-pairs test was used for non-parametric data, with *p* < 0.05 deemed significant. GM: growth medium, PMA; phorbol 12-myristate 13-acetate, IM; ionomycin

To determine the functional capacity of lymphocytes in people with ME-MM or ME-SA, we measured intracellular cytotoxic molecules (granzyme B and perforin) and cytokines (Interferon-γ (IFN-γ) and interleukin-17 (IL-17)) in T cells and NK cells. Intracellular cytokines were not detectable in ex vivo T cells (data not shown). Most NK cells contained granzyme B and perforin (granzyme B: mean 75.4% in ME-MM, 73.9% in ME-SA; perforin: 75.3% in ME-MM, 74.4% in ME-SA) (Fig. 3a, upper). Although there was no difference in the frequency of cytotoxic NK cells between the ME-MM and ME-SA groups, there was a reduced amount of granzyme B in NK cells in the ME-SA group, measured by MFI, although not of perforin (*p* = 0.025 in granzyme B expression, *p* = 0.340 in perforin) (Fig. 3a, lower). There were no differences in the frequencies of granzyme B and perforin expressing cytotoxic CD8^+^ T cells and CD4^+^ T cells between the ME-MM and ME-SA groups (Fig. 3b,upper for CD8^+^ T cells and Supplementary Figure S6 for CD4^+^ T cells). However, the MFI of both granzyme B and perforin in CD8^+^ T cells was significantly reduced in the ME-SA group (*p* = 0.021 in granzyme B expression, *p* = 0.023 in perforin) (Fig. 3b, lower). Granzyme B was detected at low frequencies in MAITs in ex vivo PBMC samples, although significantly more highly in the ME-SA than ME-MM group (mean 0.46% in ME-MM, mean 1.25% in ME-SA) (Fig. 3c, left, Fig. 3d, ‘‘ex vivo’’). However, the granzyme B-positive frequency was significantly increased after incubation for 18 hr in growth medium alone in ME-MM, but not in ME-SA (ME-MM; 2.10%, ME-SA; 1.46%, *p* = 0.002) (Fig. 3c,middle, Fig. 3d, ‘’GM’’). After in vitro culture with the stimulation cocktail comprised of PMA and ionomycin, the frequencies of intracellular granzyme B-expressing MAITs were significantly increased in both groups (*p* < 0.0001 for both), with a significantly greater up-regulation in the ME-MM group (mean 14.94%) compared to the ME-SA group (11.73%) (*p* = 0.047) (Fig. 3c, right, Fig. 3d, ‘’PMA+IM’’). A moderate proportion of MAITs expressed perforin ex vivo: the frequency of perforin-expressing MAITs was significantly lower in the ME-SA group (17.80% in ME-MM, 12.72% in ME-SA) (*p* = 0.001) (Fig. 3e, left). Furthermore, we investigated the main source of MAIT subsets which expressed perforin, and identified the CD4^-^CD8^-^ double negative MAIT subset as the major contributor, rather than the expected CD8^+^ MAIT subset, in both clinical groups (*p* < 0.001 in MM, *p* = 0.038 in SA) (Fig. 3e, middle and right). Together, these data show that CD8^+^ T cells and MAITs from people with ME-SA had a reduced capacity to express the cytotoxic mediators perforin and granzyme.

### People with ME-MM exhibited stronger correlation between cytotoxic marker expression and senescent T cells and DN MAITs

**Fig. 4:**
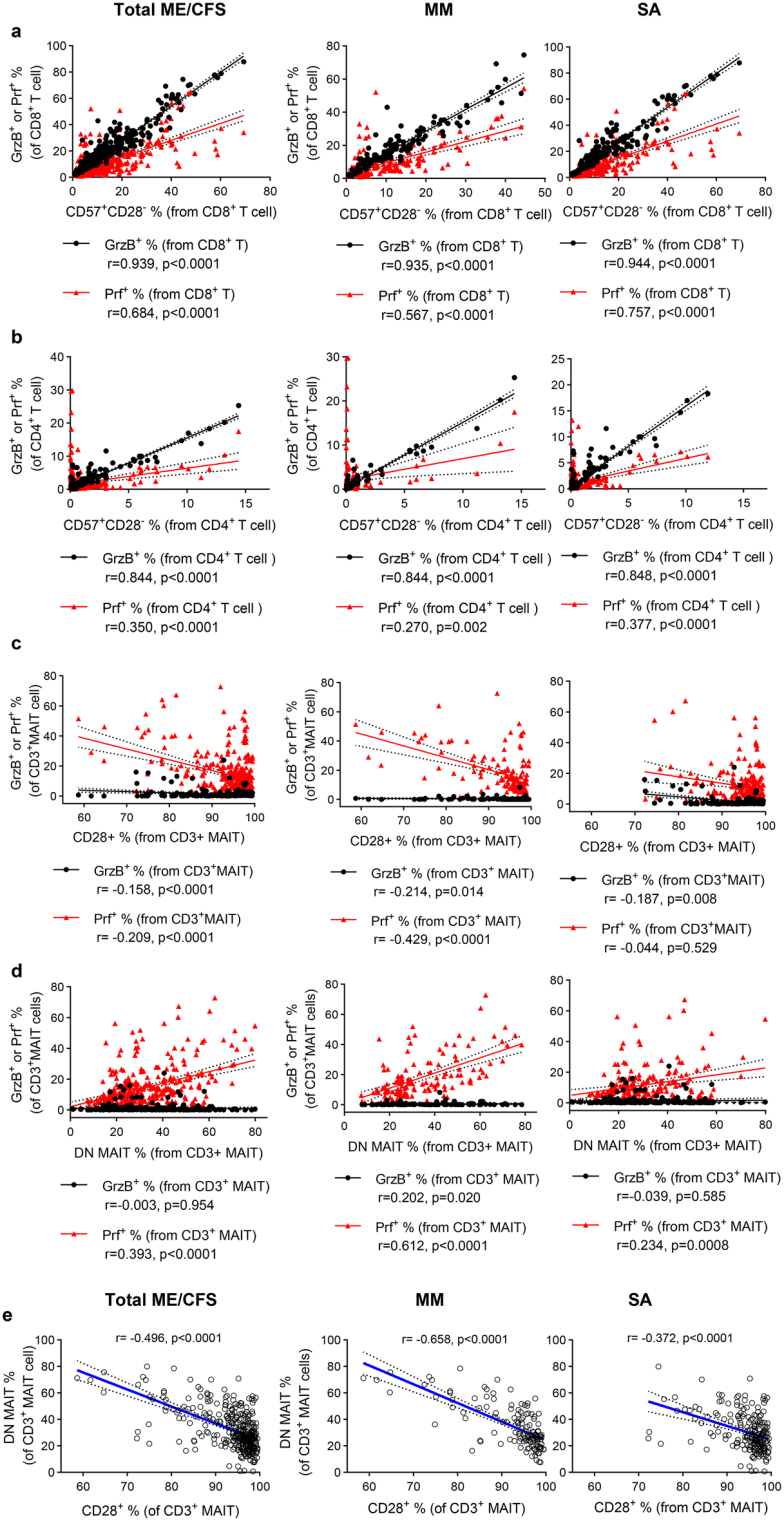
Correlation analysis between frequencies of cytotoxic T cell subsets and other T cell subsets with different differentiation status in the total sample set (*n* = 335) or by two clinical groups separately (*n* = 132 for ME-MM, *n* = 203 for ME-SA). For this analysis, the data collected from ‘memory/differentiation’ and ‘function’ flow cytometry staining panels were used. Spearman correlation analyses were performed between frequencies of (**a**) cytotoxic molecule-positive CD8^+^T cells and CD57^+^CD28^-^CD8^+^T cells, (**b**) cytotoxic molecule-positive CD4^+^T cells and CD57^+^CD28^-^CD4^+^T cell subsets, (**c**) cytotoxic molecule-positive MAITs and CD28^+^ MAITs, (**d**) cytotoxic molecule-positive MAITs and DN MAITs, and (**e**) CD28^+^MAIT cells and DN MAIT cells. Dashed lines represent the 95% confidence intervals. Each dot or triangle represents the average value across all the samples collected at different time points for individual study participants. p-value < 0.05 is deemed significant. mm: people with mild/moderate ME/CFS; SA: people severely affected with ME/CFS

To explore the potential interaction between the frequencies of cytotoxic T cell subsets and the various T cell differentiation subsets, especially the senescent CD57^+^CD28^-^ subsets, we next performed correlation analyses between the frequencies of different cell populations on all the samples (*n* = 335) or by the two clinical groups separately (*n* = 132 for ME-MM, *n* = 203 for ME-SA). Using the data acquired using the ‘memory/differentiation’ and ‘function’ flow cytometry staining panels, we found that the frequency of cytotoxic marker-positive CD8^+^ T cells was strongly correlated with the frequency of CD57^+^CD28^-^ CD8^+^ T cells (*r* = 0.939 for GrzB, *r* = 0.684 for Prf, Fig. 4a). The correlation strengths were similar in the two clinical groups. This pattern was also observed in CD4^+^ T cells, although cytotoxic effector molecules were less frequent in CD4^+^ T cells, as expected (Fig. 4b). The correlation analysis showed that samples with a lower CD28^+^ frequency in MAITs cells had a higher perforin-positive MAIT frequency (*r* = −0.209, *p* = 0.0001; Fig. 4c); furthermore, the extent of correlation was much stronger in the ME-MM group compared to the ME-SA group (*r* = −0.429, *p* < 0.0001 for MM, *r* = −0.044, *p* = 0.529 for SA; Fig. 4c). There was an even stronger association in the DN MAIT subset: the frequency of DN MAITs was positively correlated with the frequency of perforin^+^ MAIT cells across all the samples (*r* = 0.393, *p* < 0.0001 for the total sample set), while the ME-MM clinical group had higher correlation coefficients than the ME-SA (*r* = 0.612, *p* < 0.0001 for ME-MM; *r* = 0.234, *p* = 0.0008 for ME-SA; Fig. 4d). In agreement with these results, there was a negative correlation between the frequencies of CD28^+^ MAITs and DN MAITs (Fig. 4e). These findings may suggest that the samples with a higher frequency of DN MAIT cells have more cytotoxic capacity, with a more frequent appearance of the CD28^-^ population in MAITs: this is in accordance with our finding that the ME-MM group showed a non-significant trend towards higher frequency of DN MAITs (Supplementary Figure S7) as well as increased cytotoxic marker expression in MAITs and downregulation of the CD28^+^ MAIT subset.

### T cells and NK cells from people with severe ME/CFS exhibit enhanced activation status

**Fig. 5:**
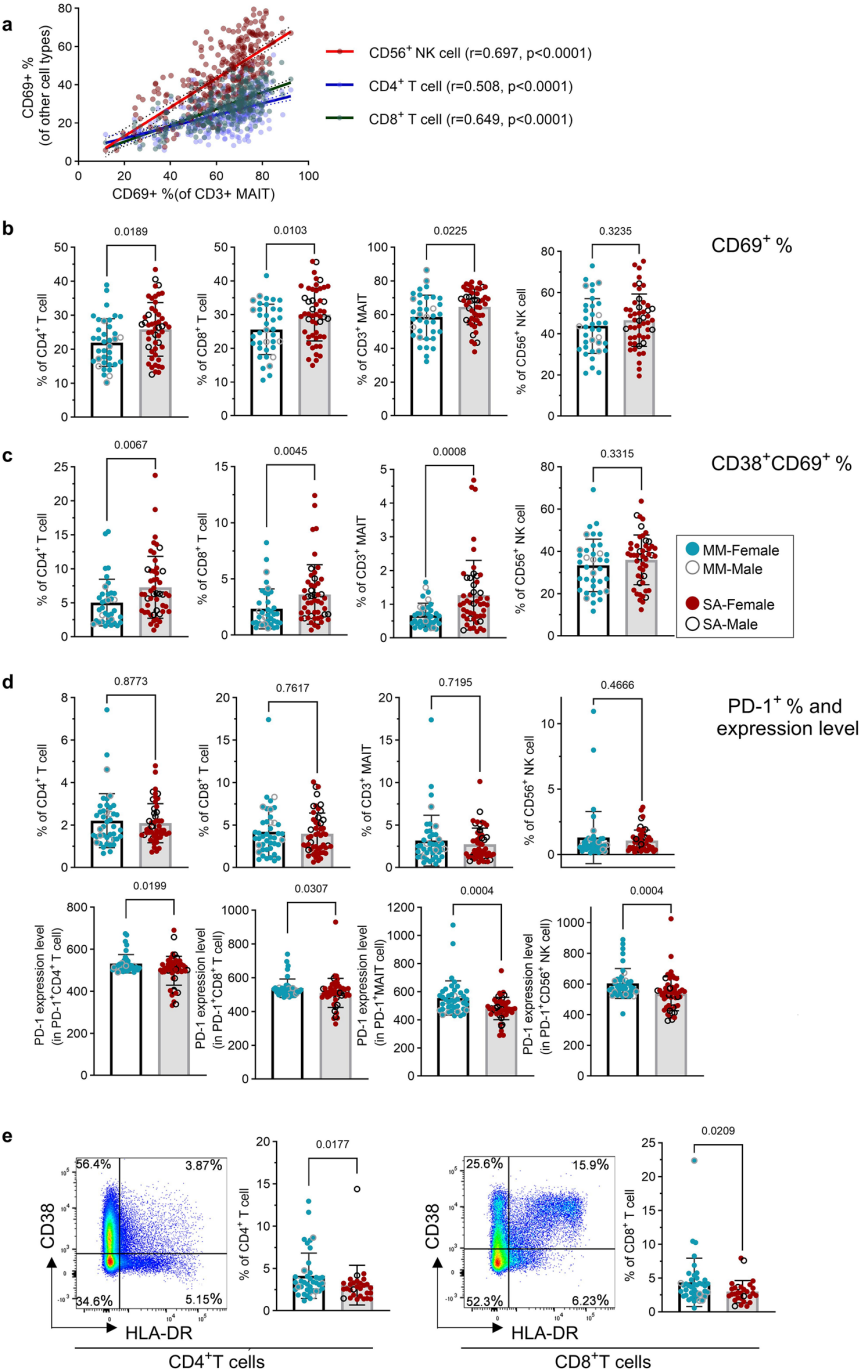
Frequencies of activation/exhaustion marker expression in T cells and NK cells from people with mild/moderate (*n* = 43) and severe ME/CFS (*n* = 53). Ex vivo PBMC were stained for surface CD69, CD38 (both as activation markers) and PD-1 (as an exhaustion marker). (**a**) Correlation analysis was performed between CD69^+^ MAITs cells and other cell types (CD69^+^ NK cells, CD69^+^CD4^+^T cells, CD69^+^CD8^+^T cells). A total of 335 samples (*n* = 132 for ME-MM, *n* = 203 for ME-SA) were analysed using the spearman correlation test. (**b**) The frequencies of CD69^+^ T cell subsets (CD4^+^ T cells, CD8^+^ T cells and MAITs) and NK cells were compared between ME-MM and ME-SA groups. (**c**) Comparison of CD38^+^CD69^+^ frequency in T cells (CD4^+^ T cells, CD8^+^ T cells and MAITs) and NK cells between ME-MM and ME-SA groups. (**d**) Comparison of PD-1 frequency in T cells and NK cells (upper) and PD-1 expression in PD-1^+^ subsets in T cells (CD4^+^ T cells, CD8^+^ T cells and MAITs) and NK cells (lower). (**e**) Representative pseudocolour plots of HLA-DR^+^CD38^+^ staining, and comparison of the frequency of HLA-DR^+^CD38^+^ T cell subsets between ME-MM (*n* = 41) and ME-SA (*n* = 29) in CD4^+^T cells (left) and CD8^+^T cells (right). Each dot represents the average value across all the samples collected at different time points for one individual. Mean values and sd are shown. The data for CD69 cells (^+^%_CD4^+^, CD8^+^ T cells and CD56+ NK cells in the first, second and fourth figures in (**b**) and CD38^+^CD69^+^ in CD56^+^ NK (fourth figure in **c**) were normally distributed and analysed by an unpaired t-test; all other datasets were not normal and were analysed by the mann-whitney test. A *p*-value of < 0.05 was considered statistically significant

Next, we investigated the expression of the activation markers CD69 and CD38, as well as the exhaustion marker, programmed death-1 (PD-1), in ex vivo PBMCs. First, we examined the association between CD69 expression on different cell types. We found a moderate and highly significant (*p* < 0.0001) correlation between the frequency of MAITs expressing CD69 and the frequency of NK cells, CD4^+^ T cells and CD8^+^ T cells expressing CD69 across all the samples (Spearman *r* = 0.697 for CD69^+^NK cells, *r* = 0.508 for CD69^+^CD4^+^ T cells, *r* = 0.649 for CD69^+^CD8^+^ T cells, Fig. 5a). Comparing the two groups, we found people with ME-SA had an elevated frequency of CD69 expression on T cell subsets, including CD4^+^ and CD8^+^ T cells and MAITs cells, compared to people with ME-MM (*p* = 0.019 in CD4^+^ T cell, *p* = 0.010 in CD8^+^ T cells, *p* = 0.023 in MAIT cells) (Fig. 5b). Moreover, the frequency of CD38^+^CD69^+^ cells within the CD4^+^ and CD8^+^ T cells and in MAITs was also significantly increased in ME-SA (*p* = 0.007 in CD4^+^ T cells, *p* = 0.005 in CD8^+^ T cells, *p* = 0.001 in MAIT cells) (Fig. 5c). Together, these data reveal a global increased activation status of lymphocytes in severe ME/CFS compared to mild/moderate illness. We also found the PD-1 expression level (MFI) from the PD-1^+^ subsets of CD4^+^ T cells, CD8^+^ T cells, MAITs and NK cells was lower in ME-SA (*p* = 0.020 in CD4^+^ T cells, *p* = 0.030 in CD8^+^ T cells, *p* = 0.0004 in MAITs, *p* = 0.0004 in NK cells) (Fig. 5d, lower), although there was no significant difference in frequency of PD-1 expressing T cells and NK cells Fig. 5d, upper. Furthermore, we also analysed another activation indicator, the co-expression of human leukocyte antigen (HLA)-DR and CD38 in CD4^+^ T cells and CD8^+^ T cells, which are considered to be expressed during viral infection [37–39]. As shown in Fig. 5e, there was a significantly higher frequency of co-expression of HLA-DR and CD38 on CD4^+^ and CD8^+^ T cells in ME-MM compared to the ME-SA group (*p* = 0.018 in CD4^+^ T cell, *p* = 0.021 in CD8^+^ T cells).

### Proinflammatory cytokine production after stimulation is elevated in ME-SA

**Fig. 6:**
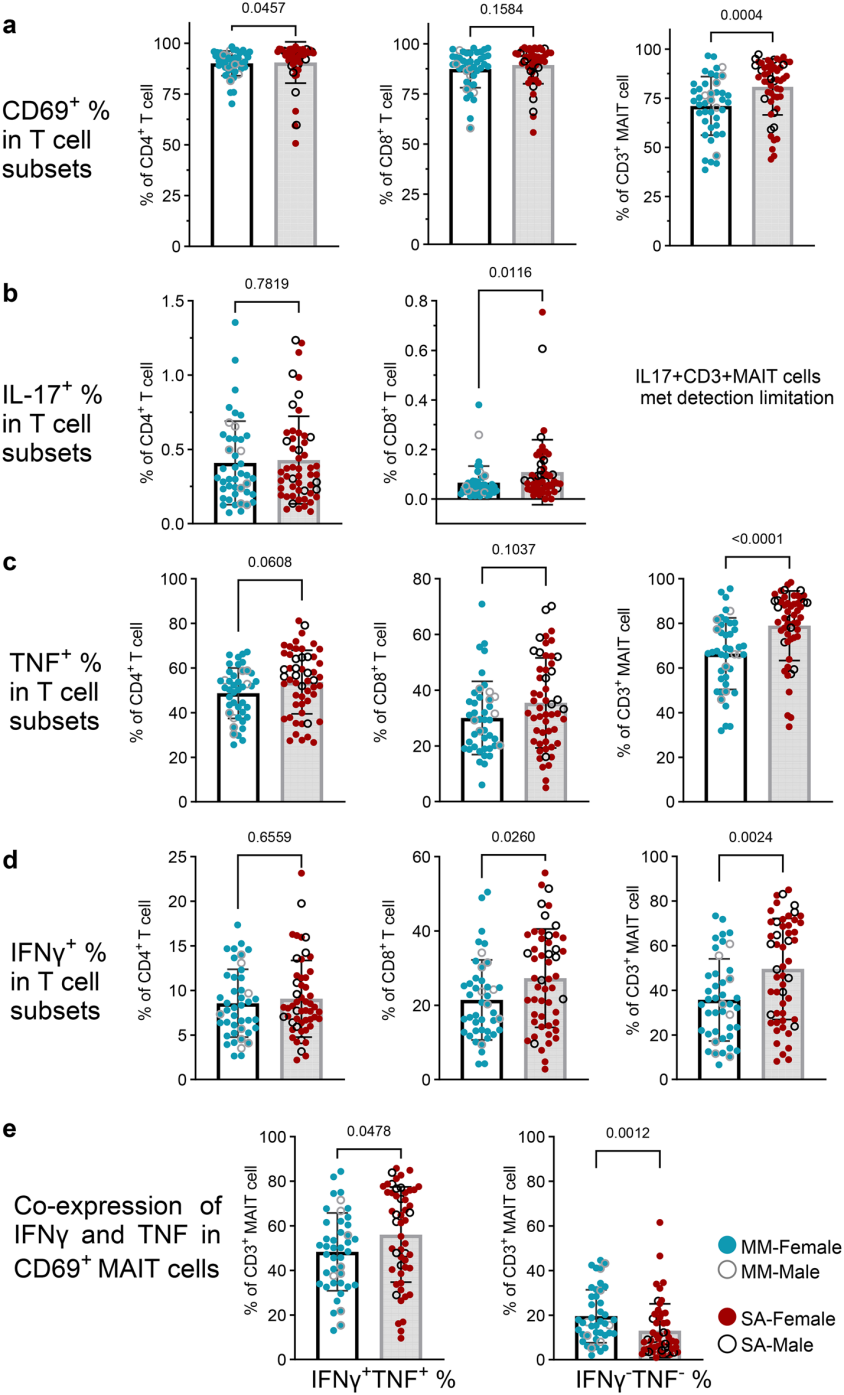
In vitro stimulation of PBMC and functional analysis of T cells from people with mild/moderate (*n* = 43) and severe ME/CFS (*n* = 53). Thawed PBMCs were incubated overnight and then stimulated with PMA and ionomycin (im) for 5 hours. Then the stimulated cells were stained for surface T cell markers (CD4, CD8, MAIT) along with the activation marker CD69, then intracellular cytokines (TNF, IFNγ and IL-17). The unstimulated cells were stained in parallel. (**a**) The CD69 expression after stimulation with PMA and IM was compared between two groups in three T cell subsets. (**b-d**) The frequencies of CD4^+^ T cells, CD8^+^ T cells and MAITs expressing IL-17 (**b**), TNF (**c**) and IFNγ (**d**) Were compared between the ME-MM and ME-SA groups. (**e**) TNF and IFNγ co-expressing CD69^+^ MAITs (left) and TNF^-^ and IFNγ^-^ non-expressing CD69^+^ MAITs were compared between the clinical groups. Each dot represents the average value across all the samples collected at different time points for one individual. Mean values and sd are shown. The data for TNF expressing (^+^%_CD4^+^ T cell, first figure in **c**) cells were normally distributed and analysed by an unpaired t-test; all other datasets were not normal and were analysed by the mann-whitney test. The dataset with *p* < 0.05 was deemed significant

We then examined the frequency of intracellular cytokine expression tumour necrosis factor (TNF), IFNγ and IL-17), along with CD69, in T cells following stimulation with PMA and ionomycin for 5 hours. As shown in Fig. 6a, most T cell subsets expressed CD69 after in vitro stimulation. The frequencies of CD69^+^CD4^+^ T cells and CD69^+^ MAITs were significantly higher in ME-SA (*p* = 0.046 in CD4^+^ T cells, *p* = 0.004 in MAITs) (Fig. 6a), consistent with the ex vivo PBMC result. Overall, IL-17 was rarely detected (Fig. 6b), however, TNF was produced by CD4^+^ T cells (48.7% in ME-MM, 53.8% in ME-SA, *p* = 0.061), CD8^+^ T cells (30.0% in ME-MM, 35.5% in ME-SA, *p* = 0.104) and MAITs (66.4% in ME-MM, 79.0% in ME-SA, *p* < 0.0001) with higher frequencies in ME-SA (Fig. 6c). The frequencies of IFNγ-producing CD8^+^ T cells and MAITs were elevated in people with ME-SA compared to ME-MM (*p* = 0.026 in CD8^+^ T cells, *p* = 0.002 in MAIT cells) (Fig. 6d). Moreover, CD8^+^ T cells from people with ME-SA had elevated frequencies of TNF, IFNγ and IL-17 production (*p* = 0.104 in TNF, *p* = 0.026 in IFNγ, *p* = 0.012 in IL-17), and MAITs from people with ME-SA had significantly elevated frequencies of TNF (*p* < 0.0001) and IFNγ (*p* = 0.002) production. Furthermore, IFNγ and TNF co-producing bifunctional MAIT cell frequency was significantly elevated in the ME-SA group (56.1%) compared to the ME-MM group (48.4%) (*p* = 0.048) (Fig. 6e), whereas the proportion of IFNγ^-^ TNF^-^ double negative MAITs was reduced (*p* = 0.001). We also found that MAIT cell activation by PMA and ionomycin changed the proportion of subsets compared to the non-stimulated condition, with an increase of DN MAITs (from 33.1% to 43.9% in ME-MM and from 23.9% to 35.1% in ME-SA) and a reduction of CD8^+^ MAITs (from 63.8% to 46.5% in ME-MM and from 74.3% to 58.9% in ME-SA) in both groups (Supplementary Figure S8).

**Fig. 7:**
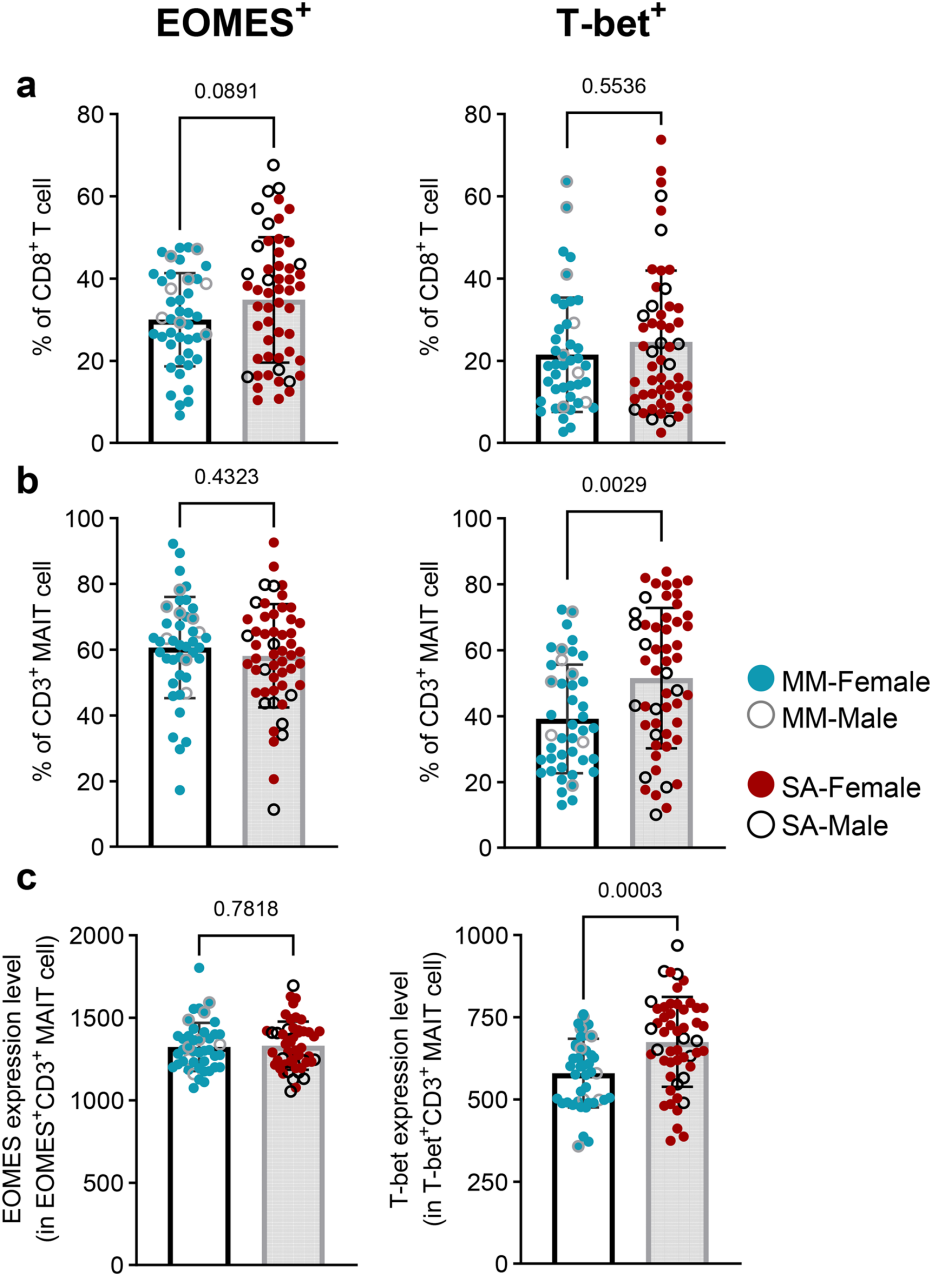
Eomes and T-bet expression in CD8^+^T and MAIT cells from people with mild/moderate (*n* = 43) and severe ME/CFS (*n* = 53). The frequency of expression of the EOMES and T-bet transcription factors was quantified by intranuclear staining after the cell phenotype extracellular staining, for CD8^+^ T cells (**a**), along with the frequency and median fluorescence intensity (MFI) in MAITs (**b,c**). Each dot represents the average value across all the samples collected at different time points for one individual. Mean values and sd are shown. The data for EOMES^+^(% in CD8^+^ T cells and CD3^+^ MAIT cells, left figures in 7a and b) cells and EOMES and T-bet MFI (in CD3^+^ MAIT cells, in 7c) were normally distributed and analysed by an unpaired t-test; all other datasets were not normal and were analysed by the mann-whitney test. The dataset with *p* < 0.05 was deemed significant

To investigate the intranuclear mechanism driving higher cytokine production in the ME-SA group, we examined the expression of transcription factors eomesodermin (EOMES) and T-box transcription factor (T-bet) in T cell subsets and NK cells in ex vivo PBMCs following intranuclear staining. EOMES and T-bet are known to drive a type 1 response, and T-bet is an essential transcription factor for optimal IFNγ production as well as NK cell maturation [40, 41]. We observed the ME-SA group had a non-significant trend towards a higher frequency of EOMES-expressing CD8^+^T cells (*p* = 0.089) and no difference in the frequency of T-bet expressing CD8^+^ T cells compared to the ME-MM group (Fig. 7a). Both the frequency and MFI of T-bet expression in MAITs were significantly increased in the ME-SA (*p* = 0.003 in frequency of T-bet, *p* = 0.0.0003 in MFI of T-bet) whereas there was no difference in EOMES expression (Fig. 7b–c). These results indicate that elevated T-bet expression in MAITs cells and more frequent EOMES expressing CD8^+^ T cells in circulating PBMC from ME-SA may play a boosting role to produce more proinflammatory cytokines after in vitro stimulation. In addition, T-bet expression level (MFI) in T-bet^+^ NK cells was significantly elevated in ME-SA (*p* = 0.044), although there was no significant difference in the frequency of T-bet^+^ NK cells because T-bet was commonly detectable in NK cells in both groups (Supplementary Figure S9).

### Cytomegalovirus seropositivity and association with cytotoxicity and senescent T cell subsets

Cytomegalovirus (CMV) latency is considered an immune confounder, affecting its host immune response in multiple ways. Therefore we measured anti-CMV immunoglobulin G (IgG) plasma concentrations to determine whether CMV seropositivity was associated with immune cell subsets. The prevalence of CMV seropositivity was similar between the two groups (MM; 30.23%, SA; 23.08%), and there was no significant difference in anti-CMV IgG concentration between the two groups although there was a wide range in both groups (Table 2). We then performed correlation analyses between CMV IgG concentration and immune cell subsets (Table 3). Notably, there was a moderate correlation between CMV IgG concentration and the frequencies of cytotoxic T cells and CD57^+^CD28^-^ subsets in CD4^+^T cells and CD8^+^ T cells in both the ME-MM and ME-SA groups, with the correlation strength higher in CD4^+^T cells than CD8^+^T cells. There was no correlation between CMV IgG concentration and cytotoxic MAIT cells or NK cells.

**Table 2:** Prevalence and concentration of CMV IgG in people with ME/CFS

	Seropositivity of CMV-IgG		CMV IgG antibody (U)		
	n	%	Average	Range (min-max)	P-value
ME-MM (*n* = 43)	13	30.23	32.63	3.16–123.90	0.9985
ME-SA (*n* = 52)	12	23.08	32.23	2.91–137.90

**Table 3:** Relationship between CMV IgG concentration and cytotoxic/senescence immune subsets

		Spearman R	95% confidence interval	p-value
ME-MM (*n* = 43)	Correlation with CMV IgG level (Units)			
	GrzB^+^CD4^+^ T cell %	**0.4047**	0.1097 to 0.6341	**0.0071**
	Prf^+^CD4^+^ T cell %	**0.5915**	0.3461 to 0.7612	** < 0.0001**
	GrzB^+^CD8^+^ T cell %	**0.4730**	0.1924 to 0.6821	**0.0014**
	Prf^+^CD8^+^ T cell %	**0.4928**	0.2171 to 0.6956	**0.0008**
	GrzB^+^ MAIT cell %	0.0171	−0.2930 to 0.3241	0.9129
	Prf^+^ MAIT cell %	0.0193	−0.2911 to 0.3260	0.9021
	GrzB^+^CD56^+^ NK cell %	0.0838	−0.2308 to 0.3826	0.5931
	Prf^+^CD56^+^ NK cell %	0.1619	−0.1545 to 0.4481	0.2997
	CD57^+^CD28^-^ CD4^+^ T cell %	**0.4825**	0.2042 to 0.6886	**0.0011**
	CD57^+^CD28^-^ CD8^+^ T cell %	**0.4063**	0.1117 to 0.6353	**0.0069**
	CD57^-^CD28^-^ CD4^+^ CM cell %	−0.0490	−0.3523 to 0.2636	0.7550
	CD57^-^CD28^-^ CD4^+^ EM cell %	0.1630	−0.1534 to 0.4490	0.2965
	CD57^-^CD28^-^ CD8^+^ EM cell %	0.1338	−0.1824 to 0.4249	0.3923
	CD57^-^CD28^-^ CD8^+^ TEMRA cell %	−0.0689	−0.3697 to 0.2449	0.6605
ME-SA (*n* = 52)	Correlation with CMV IgG level (Units)			
	GrzB^+^CD4^+^ T cell %	**0.4870**	0.2392 to 0.6753	**0.0003**
	Prf^+^CD4^+^ T cell %	**0.6281**	0.4219 to 0.7725	** < 0.0001**
	GrzB^+^CD8^+^ T cell %	**0.4885**	0.2409 to 0.6763	**0.0002**
	Prf^+^CD8^+^ T cell %	**0.3724**	0.1026 to 0.5912	**0.0066**
	GrzB^+^ MAIT cell %	0.2498	−0.03302 to 0.4956	0.0741
	Prf^+^ MAIT cell %	−0.09746	−0.3679 to 0.1882	0.4919
	GrzB^+^CD56^+^ NK cell %	−0.01656	−0.2957 to 0.2652	0.9072
	Prf^+^CD56^+^ NK cell %	−0.1723	−0.4319 to 0.1138	0.2221
	CD57^+^CD28^-^ CD4^+^ T cell %	**0.5990**	0.3828 to 0.7530	** < 0.0001**
	CD57^+^CD28^-^ CD8^+^ T cell %	**0.4903**	0.2432 to 0.6777	**0.0002**
	CD57^-^CD28^-^ CD4^+^ CM cell %	0.04893	−0.2348 to 0.3250	0.7305
	CD57^-^CD28^-^ CD4^+^ EM cell %	**0.2744**	−0.006694 to 0.5152	**0.0490**
	CD57^-^CD28^-^ CD8^+^ EM cell %	0.2230	−0.06134 to 0.4739	0.1120
	CD57^-^CD28^-^ CD8^+^ TEMRA cell %	0.1242	−0.1620 to 0.3911	0.3802

Spearman’s rank-order correlation test was used for significant correlations between two parameters of interest

### T cell activation, cytotoxicity and senescence phenotypes can discriminate mild/moderate and severe ME/CFS

**Fig. 8:**
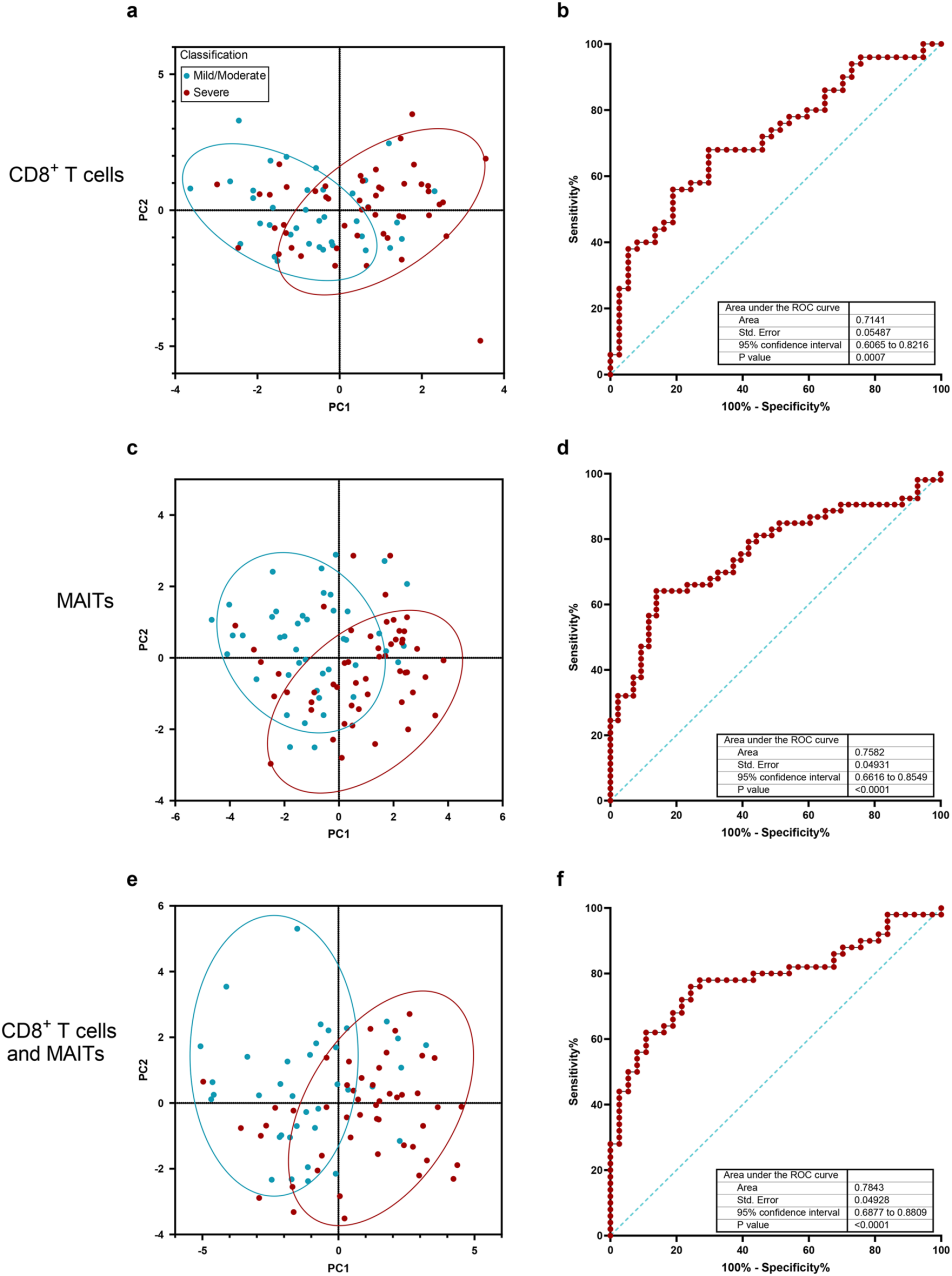
Discrimination between people with mild/moderate or severe ME/CFS based on differential expression of markers in CD8^+^ T cells and MAITs. Principal component analysis (PCA) plots of ME-MM and ME-SA study participants, based on the expression of subsets of markers in CD8^+^ T cells (**a**), MAITs (**c**) or both CD8^+^ and MAITs (**e**). Receiver operating characteristic curve analysis of ratios of activation/senescence-cytotoxicity markers in ME-SA vs ME-MM study participants in (**b**) CD8^+^ T cells ((evCD69^+^ + ivIFNγ^+^)/(CD28^-^CD57^-^CD8^+^_EM_ + CD28^-^CD57^-^CD8^+^_TEMRA_)), (**d**) MAITs ((%CD8^+^MAITs + ivTNF^+^)/(evPrf^+^ + ivGzB^+^)) or (**f**) both CD8^+^ and MAITs (((evCD69^+^ + ivIFNγ^+^)/(CD28^-^CD57^-^CD8^+^_EM_ + CD28^-^CD57^-^CD8^+^_TEMRA_)) x ((%CD8^+^MAITs + ivTNF^+^)/(evPrf^+^ + ivGzB^+^)). Parameters used in PCA analyses: CD8^+^ T cells (**a** & **e**): %CD28^-^CD57^-^CD8^+^_EM_^,^ %CD28^-^CD57^-^CD8^+^_TEMRA_, MFI-Prf evCD8^+^, MFI-GzB evCD8^+^, MFI-PD1 evCD8^+^, %evCD69^+^CD8^+^, %evCD69^+^CD38^+^CD8^+^, % ivIL-17+ CD8^+^, %ivTNF^+^CD8^+^, %ivIFNγ^+^ CD8^+^; MAITs (**c** & **e**): %MAITs, %CD8^+^MAITs, %evCD69^+^MAITs, %evCD69^+^CD38^+^MAITs, %ivCD69^+^MAITs, %ivTNF^+^MAITs %ivIFNγ^+^MAITs, %ivTNF^-^IFNγ^-^MAITs, %evT-bet^+^MAITs, %evCD28^+^MAITs, %ivGzB^+^MAITs, %evPrf^+^MAITs, MFI-PD1 evMaits. Rings in the PCA plots contain 80% of the corresponding participants. ev: ex vivo; iv: following stimulation in vitro with PMA and ionomycin

As there were significant differences observed in the expression of activation markers, proinflammatory cytokines, cytotoxicity effector molecules and immunosenescence markers between people with ME-MM and ME-SA, we next sought to determine whether combinations of these markers could be used to predict the disease severity classification. The majority of the ME-MM participants were separated from the majority of the ME-SA participants in principal component analyses based on combinations of cell markers in CD8^+^ T cells or in MAITs separately, with enhanced separation evident when the expression profiles of both CD8^+^ T cells and MAITs were combined (Fig. 8). These cell markers had moderate capacity to discriminate between the ME-MM and ME-SA clinical groups in Receiver Operating Characteristic curve analyses, with an Area Under the Curve (AUC) of 0.7141 (*p* = 0.0007) for the CD8^+^T cell markers and 0.758 (*p* < 0.0001) for the MAITs. The combination of the CD8^+^ and MAIT markers had an AUC of 0.7843 (*p* < 0.0001) (Fig. 8f), indicating that the activation, inflammation, cytotoxicity and immunosenescence markers have the capability to discriminate between people with mild/moderate or severe ME/CFS disease.

## Discussion

In this study we have demonstrated clear differences exist in immune cells in people with mild/moderate compared with severe ME/CFS, and that these differences remain over years of longitudinal sampling. It is a matter of debate whether different ME/CFS clinical severities exist on a continuum of disease progression [5, 7] or whether there are sub-groups of people with ME/CFS whose disease aetiologies and pathophysiological processes vary. There is increasing evidence of ME/CFS disease clusters, which can be derived from clinical characteristics [42, 43] or biological measurements [44]: this has implications for treatment development and personalised medicine. Our data support the existence of at least two distinct subgroups, with the sustained immunological differences suggesting different pathophysiological processes occur in mild/moderate and severe clinical groups, and that previously described differences in cross-sectional studies were not due to disease duration.

We focused on cytotoxic cell phenotype and function, including NK cells, CD8^+^ T cells and MAITs, as they have all previously been implicated in ME/CFS [12, 15, 45–48]: we addressed knowledge gaps around the functional capacity of these cells and how the cell phenotypes inter-relate. We found that people with mild to moderate ME/CFS symptoms had more evidence of early-senescence in their memory T cell subsets and MAITs, based on the loss of CD28 expression but absence of CD57 expression, within central memory CD4^+^ T cells, effector memory and TEMRA CD8^+^ T cells and lower frequencies of the CD28^+^ subset in MAITs. CD28 is a necessary costimulatory molecule for T cell activation, and the CD28^-^ T cell population is regarded as having a cytotoxic or regulatory phenotype [49]: the CD28^-^ subset in CD8^+^T cells is expanded by chronic viral infection such as human cytomegalovirus, Epstein-Barr virus and human parvovirus B19 which have been implicated in ME/CFS pathogenesis [34, 50, 51]. Using microarray analysis, Pangrazzi et al. reported that CD28^-^CD57^-^ CD8^+^ T cells showed early senescence characteristics with reduced production of IFNγ and TNF production [33]. We also observed significantly diminished CD8 staining on CD8^+^ T cells in ME-MM compared with ME-SA. CD8 down-regulation occurs transitorily in bacterial and viral infection [52] and is accompanied by enhanced cytotoxic effector function [53]. We also observed an increase in granzyme B and perforin expression in CD8^+^ T cells and NK cells in ME-MM. Taken together, these findings may suggest that the appearance of senescent and highly differentiated subsets in T cells and increased cytotoxicity as well as CD8 down-regulation could be caused by frequent exposure to antigens. In this regard, we have recently reported that human herpesviruses 6B and 7 DNA in saliva correlates with symptom severity in ME/CFS,^17^ and it is plausible that herpes virus reactivation stimulates T cells, driving them to cytotoxicity and senescence. This hypothesis should be tested, by assessment of viral reactivation and analysis of antigen-specific T cell responses.

In contrast, the early leukocyte activation marker CD69 was more highly expressed in the ME-SA group, on ex vivo CD4^+^ and CD8^+^ T cells and on MAITs, and was also more upregulated by stimulation with PMA and ionomycin in people with ME-SA than ME-MM. Thus T cells from people severely affected with ME are more readily activated both in the circulation and in vitro. Upregulated CD69-expressing immune cells in blood have been reported in autoimmune disorders, such as psoriasis and Graves’ disease [54, 55], although CD69-expressing cells have been suggested to either promote [56] or reduce disease progression [57] in models of systemic lupus erythematosus. We also observed that co-expression of another activation marker CD38 with CD69 was increased on CD4^+^ and CD8^+^ T cells and MAITs in ME-SA: a higher frequency of CD38^+^CD69^+^T cells has also been reported in acute hepatitis E virus infected patients, compared to those from resolving-phase patients [58]. Notably, increased frequencies of IFNγ-expressing cells, either alone or in combination with TNF production, were found in T cell subsets following in vitro stimulation in the ME-SA group As we have described previously [18], the ME-SA group also had elevated frequencies of circulating MAITs, more of which were CD8^+^ MAITs, whereas CD4^+^ MAIT and CD4–CD8- Double Negative (DN) MAITs were significantly lower in frequency than in the ME-MM group. MAITs are an innate-like T cell subset which play an antimicrobial role by recognising microbe riboflavin derivates presented in an MHC-related protein 1-dependent manner, to produce proinflammatory cytokines as well as cytotoxic molecules. MAIT subsets, determined by CD4 and CD8 coreceptor expression, are functionally distinct with CD8^+^ MAITs reportedly more cytotoxic with a stronger type 1 response phenotype compared to DN MAITs [59] and CD4^+^ MAITs [60]. Our data contrast to existing non-ME/CFS based literature [59], as MAITs are generally significantly reduced or depleted in blood in bacterial and viral infectious diseases, metabolic disorders, and chronic inflammatory and autoimmune diseases [61]: it is plausible that MAIT proliferative capacity [62] is enhanced in ME-SA leading to the observed elevation in blood. Alternatively, there could be dysregulation of tissue-homing chemokine receptor and integrin expression in ME-SA leading to disruption of MAIT trafficking, particularly to the small intestine; the gut microbiome composition is affected by ME/CFS [63] which may affect MAIT abundance [64]. The enhanced frequency of IFNγ^+^TNF^+^CD69^+^ CD8^+^ MAITs we discovered potentially indicates an ongoing pro-inflammatory response in ME-SA. Activated CD8^+^ MAITs are able to respond swiftly as polyfunctional effectors either TCR dependently or independently [65]. Importantly, strong correlations were observed across different cells and cell subsets, for cytotoxic effector molecules, activation markers and immunosenescence markers, indicating a systemic T/NK cell/MAIT response rather than a specific unique lymphocyte subset being affected in ME/CFS. Within the two clinical groups, there was a correlation between CMV immunoglobulin concentration and CD4^+^ and CD8^+^ T-cell cytotoxicity frequency and function: this is in accordance with the known impact of CMV on T cell differentiation, memory cell expansion and immunosuppression [66–68] which can confound immunological studies. However in this study there was no difference in CMV seropositivity or IgG concentration between the two groups, suggesting that the differences we have identified in leukocyte frequency and function are independent of CMV infection. It is plausible that another chronic viral infection, such as another member of the herpesvirus family, is exerting a different immunological effect in the two groups, and this hypothesis warrants further research. During the conduct of this study, the SARS-CoV-2 pandemic led to the emergence of Long COVID, affecting an estimated 400 million people Worldwide [69], with symptoms largely overlapping with those of ME/CFS. Immunological disturbances have also been observed in people with Long COVID [70], including persistent inflammation and dysregulated T cell responses [71], which may relate to chronic viral infection [72]. Evidence is emerging of overlapping immunological changes in Long COVID and ME/CFS [73], although the heterogeneity amongst people diagnosed with either condition means that a unifying mechanism has not been identified. Future studies should include analysis of our T cell phenotypes in sub-groups of people with Long COVID.

The strengths of our study include our focus on a very well characterised cohort. All participants completed a Symptoms Assessment form to confirm case definition compliance and study eligibility before they were accepted onto the study. Clinical assessment and additional validated questionnaires allowed further characterisation of cases by clinical phenotype and disease severity. Clinical assessment data were collected using standard equipment by an experienced research nurse trained in the study’s clinical assessment protocol. We were also able to include severely affected individuals in our study recruited using home visits: this group is often excluded from research studies. The participants provided blood samples at repeated time points, enabling us to find consistent trends between the clinical sub-groups. The study was designed to determine the immunological differences between people who are mild-to-moderately and those severely affected with ME/CFS, and a limitation of the study was that no healthy controls were included in the follow-up study. Further prospective longitudinal analyses with healthy controls will be required to facilitate the development of new treatments and diagnostic signatures, and future larger scale studies should include training, test and independent validation cohorts for biomarker verification. First, laboratory assays must be simplified and then standardised, to enable reproducible analyte measurement in small samples of fresh blood. Further characterisation will be required to determine whether measurement of cell-specific expression is required. Combining biomarkers for disease severity with other diagnostic ME/CFS biomarkers under development [74, 75] would likely lead to improved test performance, and these characteristics should be quantified in prospective multi-centre clinical trials, ideally internationally to ensure reproducibility of findings: ensuring that people with severe disease are included will be vital. Our study recruitment excluded people who were taking immunosuppressive drugs such as steroids: it is possible that over-the-counter medications such as ibuprofen could affect immune responses, and these should be controlled for in future large-scale biomarker studies. Another potential limitation was the slight sex ratio imbalance between the two clinical groups, due to the impact of the Covid-19 pandemic on participant recruitment and follow-up, which could potentially confound the analyses: nevertheless, the results we have described are consistent across both male and female participants. In this study we used phorbol ester and calcium ionophores to test the maximal responsiveness of cells from people in the two clinical groups: this might be affected by prior in vivo activation and in future, more physiologically relevant stimuli such as viral antigens should be included to further characterise T cell responsiveness.

## Conclusions

It is still not well established if mild/moderate and severe cases of ME/CFS represent a spectrum of clinical phenotypical expressions of the same pathophysiological processes, or if they rather largely relate to distinct processes. With a paucity of studies investigating more severely affected people living with ME/CFS, our study adds important information on immunological differences between these groups indicative of different disease aetiology and pathogenesis mechanisms in the two groups. These findings may suggest that people with ME-MM have frequent antigen exposure, which might be related to persistent viral infection and frequent reactivation, leading to the appearance of early senescence memory T cells and MAIT cells, and also the expression of more cytotoxic effector molecules. In contrast, people with ME-SA had evidence of an ongoing uncontrolled pro-inflammatory immune system activation, with more activated T cells in blood, and higher cell activation and pro-inflammatory cytokine production in response to stimulation. The sustained activation and inflammatory cytokine production in ME-SA may be a cause or result of symptom exacerbation, and may contribute to symptom severity. Our results also suggest that biomarkers can be developed which classify to which sub-group a person with ME/CFS belongs, to be used to stratify patients for prognosis and clinical management, and to aid treatment choices when new therapies become available [76–80].

## Electronic supplementary material

Below is the link to the electronic supplementary material.

Supplementary Material 1

Supplementary Material 2

Supplementary Material 3

Supplementary Material 4

Supplementary Material 5

Supplementary Material 6

Supplementary Material 7

Supplementary Material 8

Supplementary Material 9

Supplementary Material 10

Supplementary Material 11

Supplementary Material 12

## Data Availability

All data generated and analysed during this study are included as Additional File 14.

## Abbreviations

AUC: Area under the curve
CCR7: C-C Chemokine Receptor 7
CM: Central Memory
CMV: Cytomegalovirus
DN: Double negative (CD4CD8 T cells)
DP: Double Positive (CD4^+^CD8^+^ T cells)
EM: Effector Memory
EOMES: Eomesodermin
FBS: Foetal Bovine Serum
FMO: Fluorescence Minus One
GzB: Granzyme B
HLA-DR: Human Leukocyte Antigen-DR
IFNγ: Interferon-γ
IL-17: Interleukin-17
IM: Ionomycin
LSHTM: London School of Hygiene & Tropical Medicine
MAITs: Mucosal-Associated Invariant T Cells
ME/CFS: Myalgic Encephalomyelitis/Chronic Fatigue Syndrome
ME-MM: Mild-to-moderately affected with ME/CFS
ME-SA: Severely affected with ME/CFS
MFI: Median Fluorescence Intensity
MR1: MHC class I-related protein 1
NK: Natural Killer
NK8: CD8+ NK cell
NKT: Natural Killer T Cell
PBMCs: Peripheral Blood Mononuclear Cells
PCA: Principal Component Analysis
PD1: Programmed Death-1
PEM: Post-exertional malaise
PLZF: Promyelocytic Leukemia Zinc Finger Protein
PMA: Phorbol 12-Myristate 13-Acetate
Prf: perforin
PWME: People living with ME/CFS
ROC: Receiver Operating Characteristic
RORγt: Retinoic acid-related Orphan Receptor gamma t
T-bet: T-box transcription factor,
TCR: T Cell Receptor
TEMRA: Terminally Re-expressing CD45RA Effector Cells
TIM-3: T cell Immunoglobulin and Mucin domain containing protein 3,
TNF: Tumour Necrosis Factor
