# Supplementary material for: Abnormal T-Cell activation and cytotoxic T-Cell frequency discriminate symptom severity in myalgic encephalomyelitis/chronic fatigue syndrome
Source: J Transl Med. 2025 Dec 10;24:68. doi: 10.1186/s12967-025-07507-x (PMC12801500; doi:10.1186/s12967-025-07507-x)
Supplement: Supplementary file 1 — Supplementary Material 1 [file 12967_2025_7507_MOESM1_ESM.pdf]

Supplementary Table S1: Fluorescently labelled antibodies used for flow cytometry

Name	fluorochrome	manufacturer	clone	isotype	catalog number	RRID
CD3	AF700	ebioscience	UCHT1	Mouse IgG1, κ	56-0038-82	AB_906220
CD4	V500	BD Horizon	RPA-T4	Mouse IgG1, κ	560768	AB_1937323
CD4	PE	BioLegend	RPA-T4	Mouse IgG1, κ	300507	AB_314075
CD8	BV711	BioLegend	RPA-T8	Mouse IgG1, κ	301044	AB_2562906
CD8	PE-Cy7	BioLegend	HIT8a	Mouse IgG1, κ	300913	AB_314117
CD56	BV650	BioLegend	HCD56	Mouse IgG1, κ	318344	AB_2563838
MR1 5-OP-RU tetramer, MR1 6-FP tetramer (negative control)	PE	Gifted from NIH Tetramer Core Facility				
CD161	PerCP-Cy5.5	ebioscience	HP-3G10	Mouse IgG1, κ	45-1619-42	AB_1311148
TCR Vα7.2	BV605	BioLegend	3C10	Mouse IgG1, κ	351720	AB_2563991
Live/dead/ Near-IR	APC/Cy7	ebioscience			L34976	
CCR7	APC	Biolegend	G043H7	Mouse IgG2a, κ	353214	AB_10917387
CD45RA	BB515	BD Horizon	HI100	Mouse IgG2b, κ	564552	AB_2738841
CD28	PerCP-Cy5.5	Biolegend	CD28.2	Mouse IgG1, κ	302922	AB_2073718
CD57	eFluor 450	eBioscience	TB01	Mouse IgM	48-0577-42	AB_2016680
PD-1	FITC	BioLegend	EH12.2H7	Mouse IgG1, κ	329904	AB_940479
PD-1	BV650	BioLegend	EH12.2H7	Mouse IgG1, κ	329950	AB_2566362
CD69	APC	BioLegend	FN50	Mouse IgG1, κ	310910	AB_314845
CD69	PE-Cy7	eBioscience	FN50	Mouse IgG1, κ	25-0699-42	AB_1548714
CD38	PE-eFluor 610	eBioscience	HIT2	Mouse IgG1, κ	61-0389-42	AB_2574552
CD38	BV510	BioLegend	HB-7	Mouse IgG1, κ	356612	AB_2563875
TIM-3	BV421	BioLegend	F38-2E2	Mouse IgG1, κ	345008	AB_11218598
PLZF	AF488	eBioscience	Mags.21F7	Mouse IgG1, κ	53-9320-82	AB_2574445
T-bet	eFlour660	eBioscience	eBio4B10 (4B10)	Mouse IgG1, κ	50-5825-82	AB_10596655
EOMES	PE-eFluor 610	eBioscience	WD1928	Mouse IgG1, κ	61-4877-42	AB_2574616
RORγt	BV421	BD Horizon	Q21-559	Mouse IgG2b, κ	563282	AB_2738114
GranzymeB	FITC	BioLegend	GB11	Mouse IgG1, κ	515403	AB_2114575
IL17A	eFlour660	eBioscience	eBio64CAP17	Mouse IgG1, κ	50-7178-42	AB_2574282
IFNγ	PE-eFluor 610	eBioscience	4S.B3	Mouse IgG1, κ	61-7319-42	AB_2574664
Perforin	BV421	BioLegend	B-D48	Mouse IgG1, κ	353307	AB_11149688
TNF	eFluor 450	eBioscience	MAb11	Mouse IgG1, κ	48-7349-42	AB_2043889
HLA-DR	BV421	BioLegend	L243	Mouse IgG2a, κ	307636	AB_2561831

MR1: MHC class I-related protein 1, CCR7:C-C chemokine receptor 7, PD-1:programmed death-1, TIM-3:T cell immunoglobulin and mucin domain containing protein 3, PLZF: promyelocytic leukemia zinc finger protein, EOMES: eomesodermin, T-bet: T-box transcription factor, RORγt: retinoic acid-related orphan receptor gamma t, IFNγ: interferon gamma, TNF: tumour necrosis factor, HLA-DR: human leukocyte antigen-DR
