# Supplementary material for: Abnormal T-Cell activation and cytotoxic T-Cell frequency discriminate symptom severity in myalgic encephalomyelitis/chronic fatigue syndrome
Source: J Transl Med. 2025 Dec 10;24:68. doi: 10.1186/s12967-025-07507-x (PMC12801500; doi:10.1186/s12967-025-07507-x)
Supplement: Supplementary file 2 — Supplementary Material 2 [file 12967_2025_7507_MOESM2_ESM.pdf]

Supplementary Table S2: Flow cytometry panel descriptions

	Ex vivo					*In vitro*
	**Panel 1**	**Panel 2**	**Panel 3**	**Panel 4**	**Panel 5**	**Panel 6**
**Fluorochromes**	**Activation/ Exhaustion**	**Memory/ differentiation**	**Function**	**Transcription Factor**	**Combined panels 1 to 4**	**Function**
AF700	CD3	CD3	CD3	CD3	CD3	CD3
V500 or BV510	CD4	CD4	CD4	CD4	CD38	CD4
BV711	CD8	CD8	CD8	CD8	Perforin	CD8
BV650	CD56	CD56	CD56	CD56	PD-1	PD-1
BV605	TCR Vα7.2	TCR Vα7.2	TCR Vα7.2	TCR Vα7.2	T-bet	TCR Vα7.2
PerCP-Cy5.5	CD161	CD28	CD161	CD161	Granzyme B	CD161
PE	MR1 5-OP-RU+	MR1	MR1	MR1	CD4	MR1
Fixable Near-IR	Viability	Viability	Viability	Viability	Viability	Viability
FITC or BB515 or AF488	PD-1	CD45RA	Granzyme B	PLZF	CD45RA	Granzyme B
APC or Ef660	CD69	CCR7	IL-17	T-bet	CCR7	IL-17
PE-eFluor610	CD38		IFNγ	EOMES	EOMES	IFNγ
BV421 or eFlour450	TIM-3	CD57	Perforin	RORγt	HLA-DR	TNFα
PE-Cy7					CD8	CD69
