# Supplementary material for: Abnormal T-Cell activation and cytotoxic T-Cell frequency discriminate symptom severity in myalgic encephalomyelitis/chronic fatigue syndrome
Source: J Transl Med. 2025 Dec 10;24:68. doi: 10.1186/s12967-025-07507-x (PMC12801500; doi:10.1186/s12967-025-07507-x)
Supplement: Supplementary file 3 — Supplementary Material 3 [file 12967_2025_7507_MOESM3_ESM.pdf]

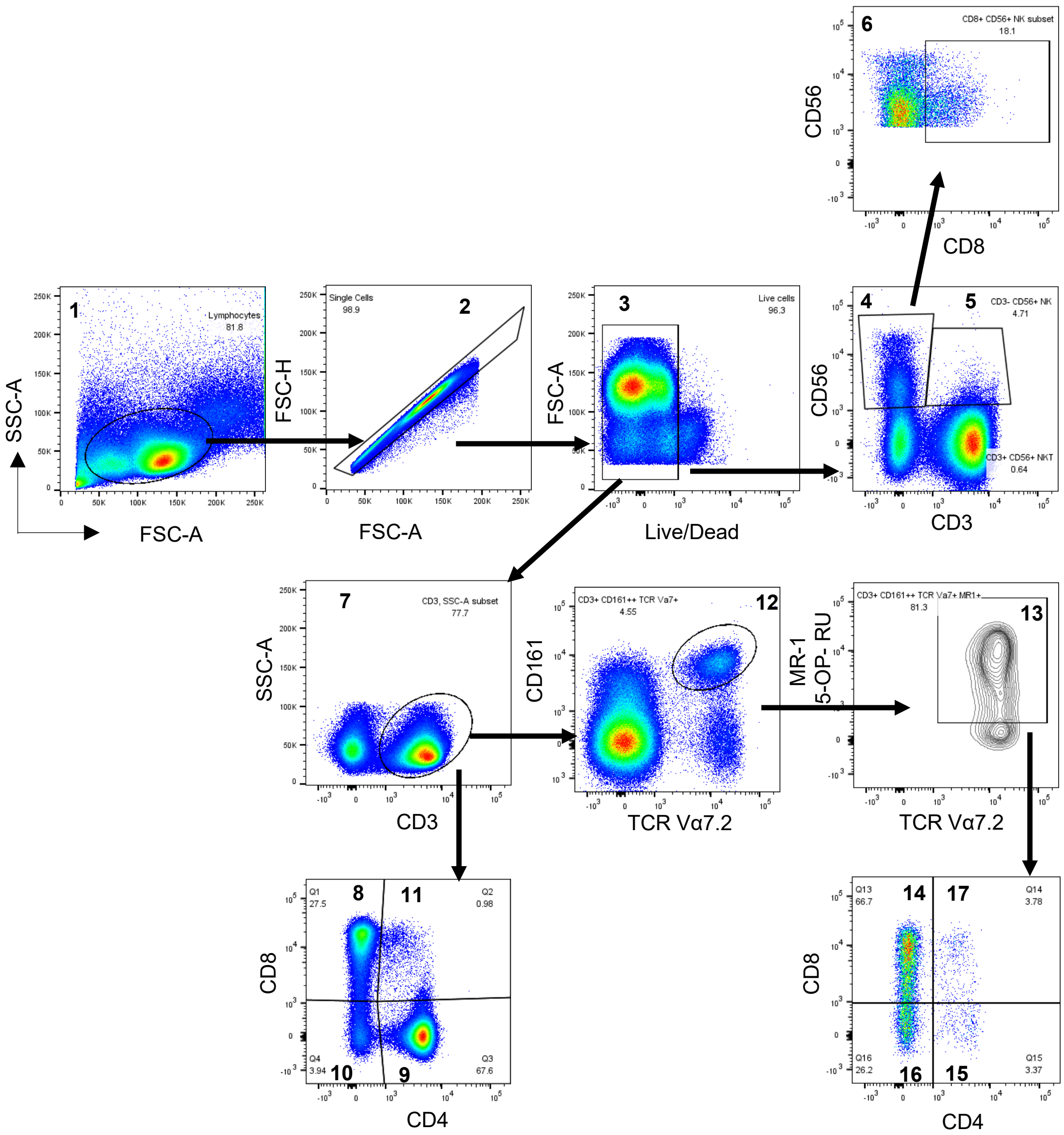

Supplementary Figure S1: Lymphocyte gating strategy for phenotyping of PBMC. Thawed PBMC from people with ME/CFS were stained with fluorescently labelled antibodies (Supplementary Table S1). Data were collected by flow cytometry and analysed using FlowJo software. (1) Lymphocytes were identified by forward and side scatter area profile, (2) singlet cells were identified by forward scatter height and area, and the other cell types were identified as positive populations based on gating by FMO controls and MR-1 tetramer loaded with 6-FP (as a negative control of MR-1 tetramer with 5-OP-RU). **(1)** lymphocytes, **(2)** singlet, **(3)** live cells, **(4)** Natural killer cells (NK cells) as $CD3^-CD56^+$, **(5)** Natural killer T (NKT)-like cells as $CD3^+CD56^+$, **(6)** $CD8^+$ NK cells (NK8 cells) as $CD3^-CD56^+CD8^+$, **(7)** T cells as $CD3^+$. Within the T cell gate, T cells were characterised further based on CD4 and CD8 expression: **(8)** $CD8^+$T cells, **(9)** $CD4^+$T cells, **(10)** $CD4^-CD8^-$ double negative (DN) cells, **(11)** $CD4^+CD8^+$ double positive (DP) cells. In parallel, the T cell gate was characterised by expression of TCR Vα7.2 and CD161. **(12)** $CD3^+$TCR Vα7.2^+CD161^{++} cells (generally recognised as mucosal-associated invariant T cells (MAIT cells), **(13)** In this study, MAIT cells were defined as $CD3^+$TCR Vα7.2^+CD161^{++}MR-1^+ tetramer (loaded with 5-OP-RU). Further, the MAIT cells were characterised based on expression of CD4 and CD8: **(14)** $CD8^+$ MAIT cells, **(15)** $CD4^+$ MAIT cells, **(16)** $CD4^-CD8^-$ DN MAIT cells, **(17)** $CD4^+CD8^+$ DP MAIT cells. For the proper gating of immune cells, we used fluorescence minus one (FMO) for CD3, CD4, CD8, CD161, TCR Vα7.2 and CD56 and MR1 tetramer loaded with 6-FP as a negative control . This figure was a representative example obtained using the 'activation/exhaustion' staining panel. MR-1: MHC class I-related protein 1
