# Supplementary material for: Abnormal T-Cell activation and cytotoxic T-Cell frequency discriminate symptom severity in myalgic encephalomyelitis/chronic fatigue syndrome
Source: J Transl Med. 2025 Dec 10;24:68. doi: 10.1186/s12967-025-07507-x (PMC12801500; doi:10.1186/s12967-025-07507-x)
Supplement: Supplementary file 4 — Supplementary Material 4 [file 12967_2025_7507_MOESM4_ESM.pdf]

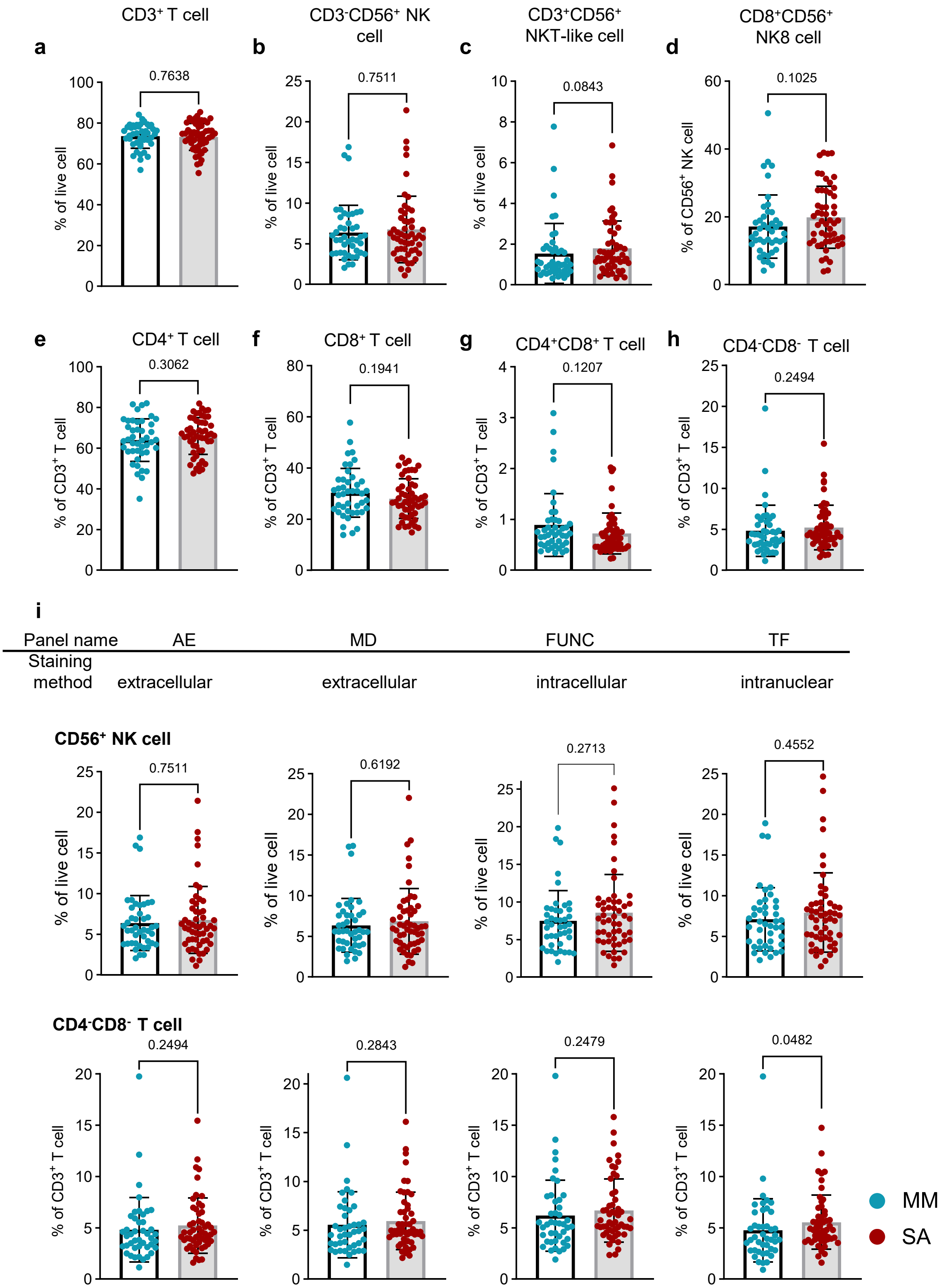

Supplementary Figure S2: Comparison of frequencies of T cells and NK cells and their subsets in people with mild/moderate (n=43) and severe ME/CFS (n=53). The frequencies of **(a)** T cells, **(b)** NK cells, **(c)** NKT-like cells, **(d)** $CD8^+CD56^+$NK8 cells, **(e)** $CD4^+$T cells, **(f)** $CD8^+$T cells, **(g)** $CD4^-CD8^-$T cells, and **(h)** $CD4^+CD8^+$T cells within parent populations (shown on y-axis) were compared between the two clinical groups, using data obtained from the 'activation/exhaustion marker' staining panel. **(i)** Frequencies of NK cells and $CD4^-CD8^-$ DN T cells in *ex vivo* PBMC stained by using different panels are shown. "AE": Activation/Exhaustion markers; ''MD'': Memory/Differentiation; "Func": functional markers; "TF": transcription factor markers. Each dot represents the average value across all the samples collected at different time points for individual study participants. Mean values and SD for each group are shown. Data were compared using the Mann-Whitney test for non-parametric data, with p<0.05 deemed significant. MM: people with mild/moderate symptoms, SA; severely affected people.
