# Supplementary material for: Abnormal T-Cell activation and cytotoxic T-Cell frequency discriminate symptom severity in myalgic encephalomyelitis/chronic fatigue syndrome
Source: J Transl Med. 2025 Dec 10;24:68. doi: 10.1186/s12967-025-07507-x (PMC12801500; doi:10.1186/s12967-025-07507-x)
Supplement: Supplementary file 5 — Supplementary Material 5 [file 12967_2025_7507_MOESM5_ESM.pdf]

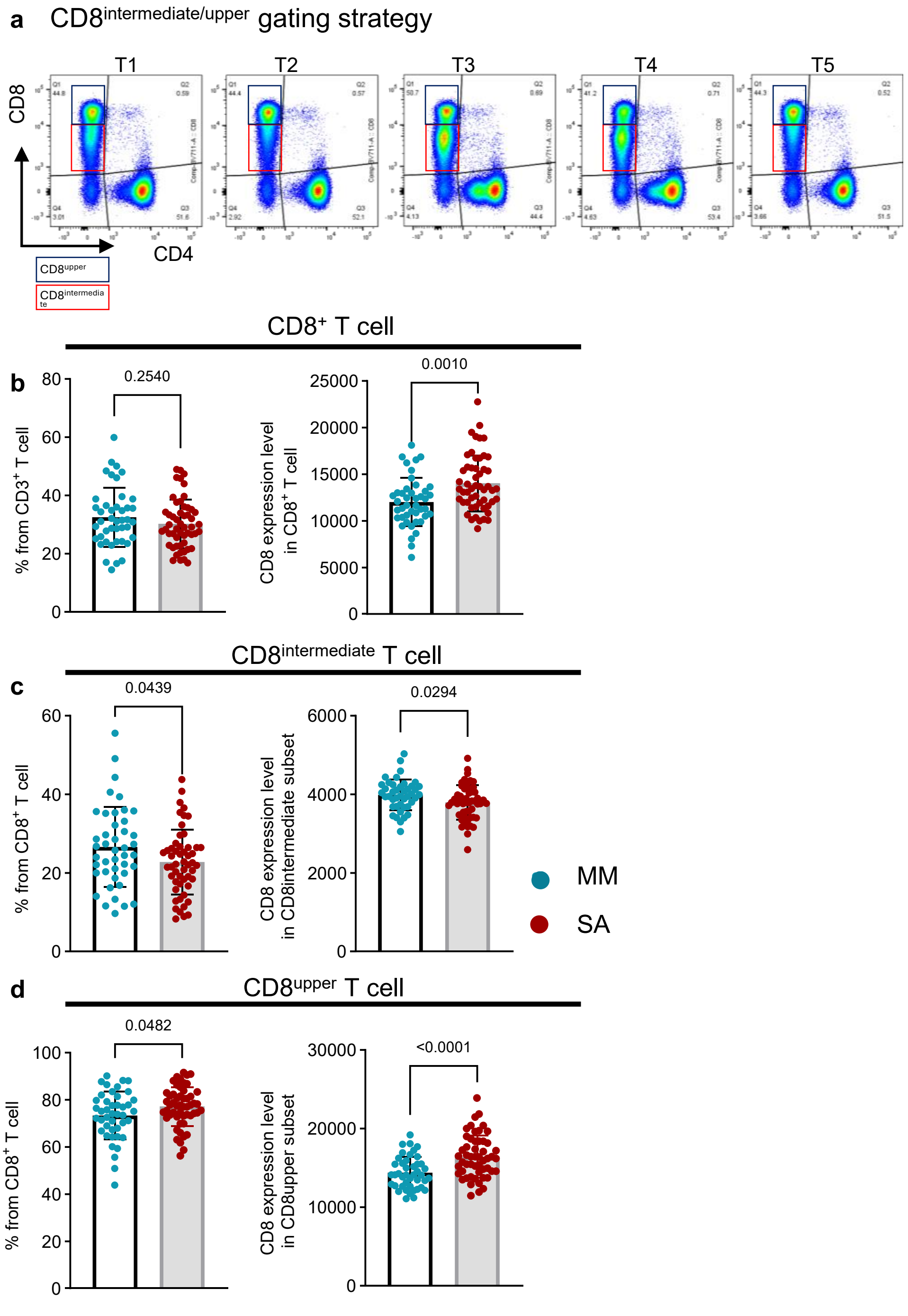

Supplementary Figure S3: Differential median fluorescence intensity of CD8α in people with mild/moderate (n=43) and severe ME/CFS (n=53). (a) The $CD8^{intermediate}$ and $CD8^{upper}$ population within the CD8 population was gated, based on a sample that has distinct upper and intermediate populations e.g T3 and T4. Gating for $CD8^{intermediate/upper}$ population is shown as a representative pseudocolour plot. **(b)** The frequency of $CD8^{+}$ T cells (left) and median fluorescence intensity of CD8α (right) were compared between the two groups. The dataset was from 'functional marker' staining panel. **(c)** The frequency of $CD8^{intermediate}$ population and CD8 MFI in the $CD8^{intermediate}$ population was compared between two groups **(d)** The frequency of $CD8^{upper}$ population and CD8 MFI in the $CD8^{upper}$ population was compared between two groups. Each dot represents the average value across all the samples collected at different time points for individual study participants. Mean values and SD are shown. Datasets were compared using the Mann-Whitney test for non-parametric data or the t-test for parametric data, with $p<0.05$ deemed significant. MM: people with mild/moderate symptoms; SA: severely affected people. T: time
