# Supplementary material for: Abnormal T-Cell activation and cytotoxic T-Cell frequency discriminate symptom severity in myalgic encephalomyelitis/chronic fatigue syndrome
Source: J Transl Med. 2025 Dec 10;24:68. doi: 10.1186/s12967-025-07507-x (PMC12801500; doi:10.1186/s12967-025-07507-x)
Supplement: Supplementary file 6 — Supplementary Material 6 [file 12967_2025_7507_MOESM6_ESM.pdf]

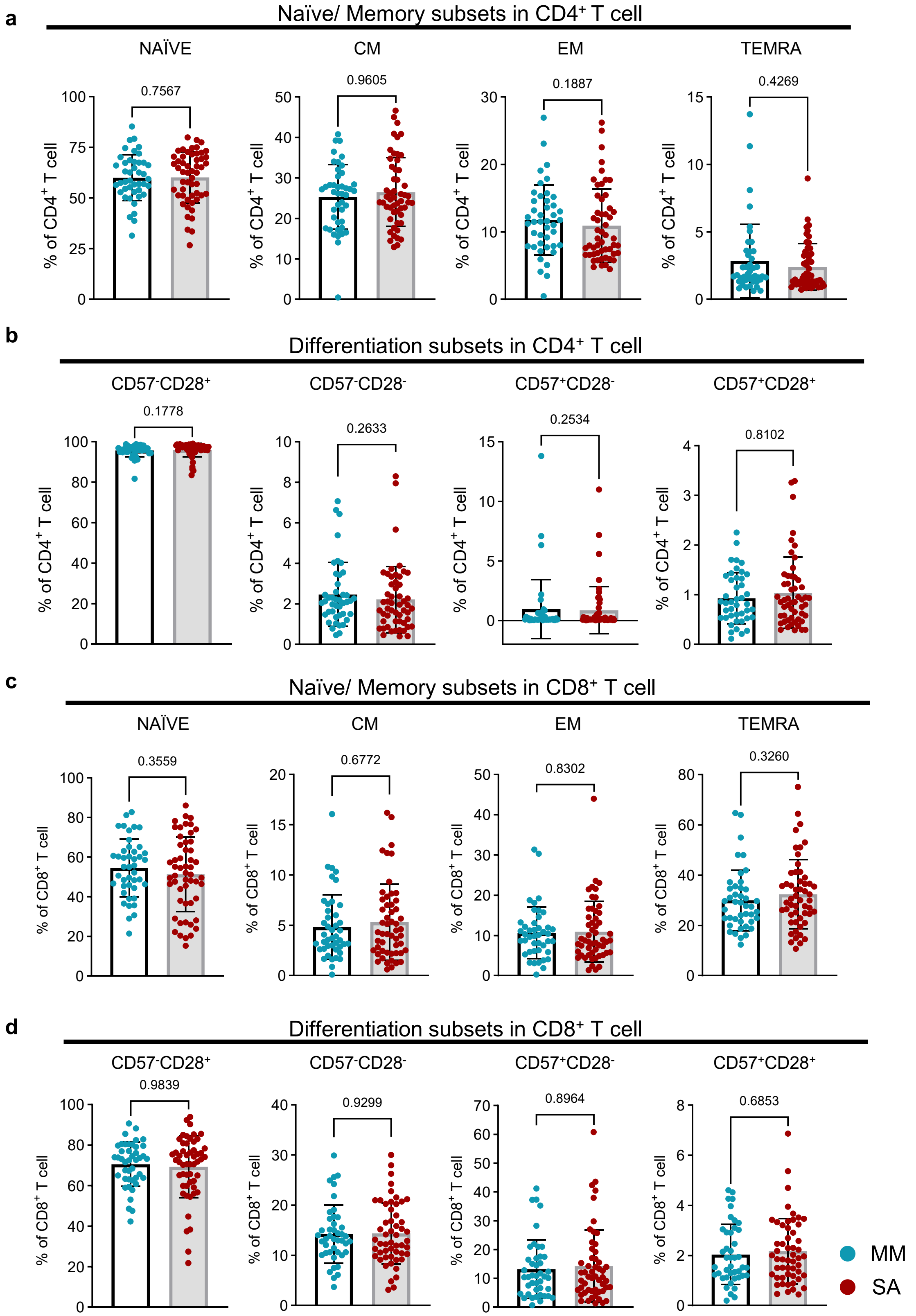

Supplementary Figure S4: Frequencies of naïve/memory and differentiation subsets in CD4$^+$ T cells and CD8$^+$ T cells from people with mild/moderate (n=43) and severe ME/CFS (n=53). *Ex vivo* PBMC were analysed using the 'memory/differentiation panel', comprised of CD45RA, CCR7, CD57, CD28. Within the CD3$^+$T cell compartment, CD4$^+$ and CD8$^+$T cells were analysed by expression of CCR7 and CD45RA to define naïve and memory cells **(a and c)** and CD57 and CD28 to define differentiation status **(b and d)**. The frequencies were compared between the two clinical groups. Naïve: CCR7$^+$CD45RA$^+$; Central Memory (CM): CCR7$^+$CD45RA$^-$; Effector Memory (EM):CCR7$^-$CD45RA$^-$; terminally re-expressing CD45RA effector cells (TEMRA): CCR7$^-$CD45RA$^+$. Each dot represents the average value across all the samples collected at different time points for individual study participants. Mean values and SD are shown. Datasets were compared using the Mann-Whitney test for non-parametric data, with p<0.05 deemed significant. MM: people with mild/moderate symptoms; SA: severely affected people.
