# Supplementary material for: Abnormal T-Cell activation and cytotoxic T-Cell frequency discriminate symptom severity in myalgic encephalomyelitis/chronic fatigue syndrome
Source: J Transl Med. 2025 Dec 10;24:68. doi: 10.1186/s12967-025-07507-x (PMC12801500; doi:10.1186/s12967-025-07507-x)
Supplement: Supplementary file 7 — Supplementary Material 7 [file 12967_2025_7507_MOESM7_ESM.pdf]

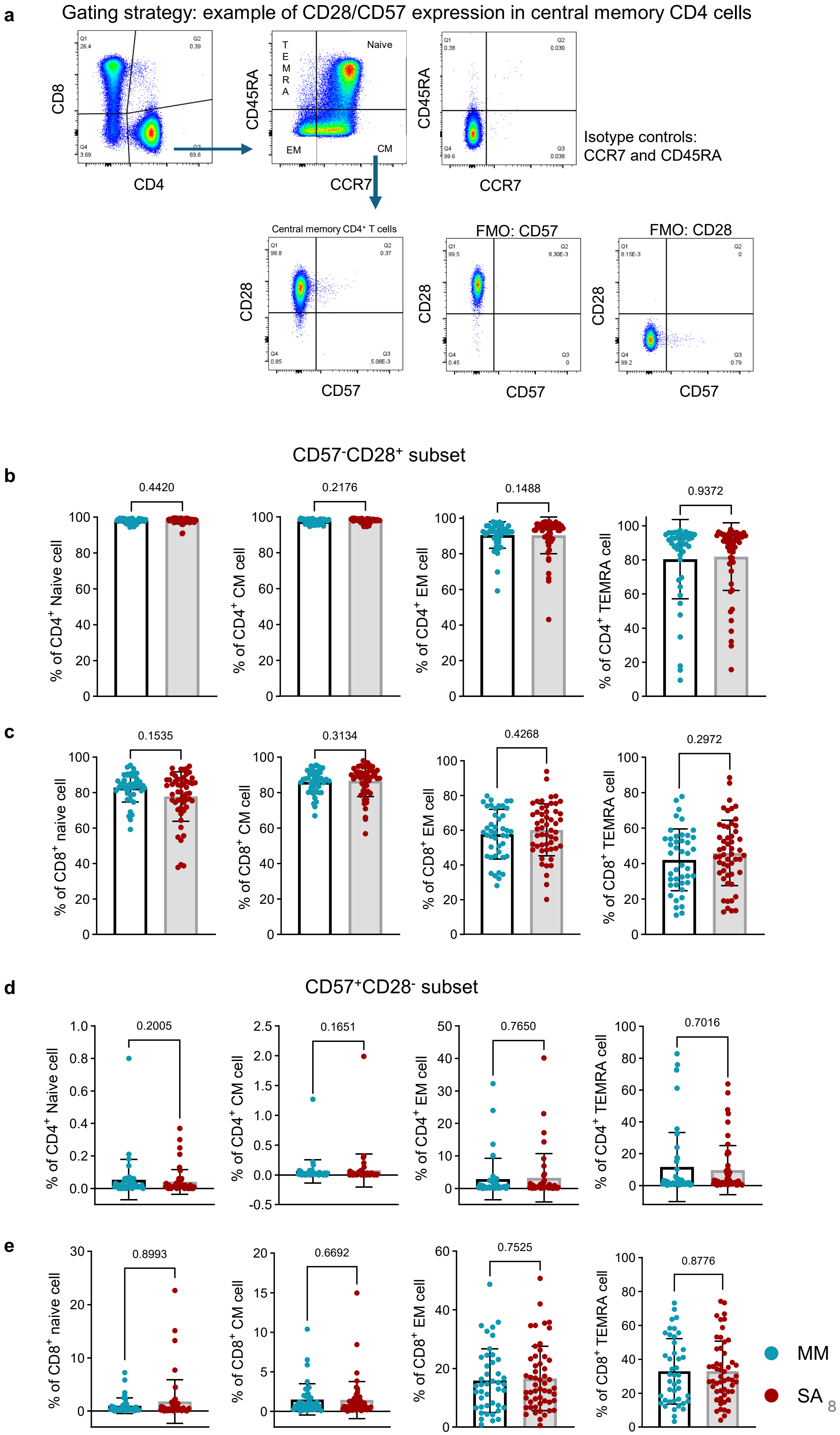
a
Gating strategy: example of CD28/CD57 expression in central memory CD4 cells
CD8
CD4
CD45RA
CCR7
TEMRA
Naive
EM
CM
Isotype controls:
CCR7 and CD45RA
Central memory CD4⁺ T cells
FMO: CD57
FMO: CD28
CD28
CD57
CD57⁻CD28⁺ subset
b
0.4420
0.2176
0.1488
0.9372
% of CD4⁺ Naive cell
% of CD4⁺ CM cell
% of CD4⁺ EM cell
% of CD4⁺ TEMRA cell
c
0.1535
0.3134
0.4268
0.2972
% of CD8⁺ naive cell
% of CD8⁺ CM cell
% of CD8⁺ EM cell
% of CD8⁺ TEMRA cell
CD57⁺CD28⁻ subset
d
0.2005
0.1651
0.7650
0.7016
% of CD4⁺ Naive cell
% of CD4⁺ CM cell
% of CD4⁺ EM cell
% of CD4⁺ TEMRA cell
e
0.8993
0.6692
0.7525
0.8776
% of CD8⁺ naive cell
% of CD8⁺ CM cell
% of CD8⁺ EM cell
% of CD8⁺ TEMRA cell
MM
SA

**Supplementary Figure S5: Analysis of naïve /memory subsets of CD4$^+$ and CD8$^+$ T cells by expression of CD57 and CD28 in people with mild/moderate (n=43) and severe ME/CFS (n=53).** *Ex vivo* PBMC were analysed using the 'memory/differentiation' staining panel, comprised of CD45RA, CCR7, CD57, and CD28. Each CD4$^+$ and CD8$^+$ naïve/memory T cell subset was further analysed for expression of CD57 and CD28, to define CD57$^-$CD28$^+$, CD57$^-$CD28$^-$, CD57$^+$CD28$^-$ and CD57$^+$CD28$^+$ subsets. **(a)** Example of the gating strategy of CD57 and CD28 populations in memory CD4$^+$ T cells. We used isotype controls for CCR7 and CD45RA, and fluorescence minus one (FMO) for CD28 and CD57 in each experiment. The frequencies of the CD57$^-$CD28$^+$ subset **(b and c)** and the CD57$^+$CD28$^-$ subset **(d and e)** are shown for naïve, CM, EM and TEMRA CD4$^+$ **(b and d)** and CD8$^+$ **(c and e)** T cells. Each dot represents the average value across all the samples collected at different time points for individual study participants. Mean values and SD are shown, and datasets were compared using the Mann-Whitney test for non-parametric data or the unpaired t-test for parametric data, with $p<0.05$ deemed significant. MM: people with mild/moderate symptoms; SA: severely affected people.
