# Supplementary material for: Abnormal T-Cell activation and cytotoxic T-Cell frequency discriminate symptom severity in myalgic encephalomyelitis/chronic fatigue syndrome
Source: J Transl Med. 2025 Dec 10;24:68. doi: 10.1186/s12967-025-07507-x (PMC12801500; doi:10.1186/s12967-025-07507-x)
Supplement: Supplementary file 8 — Supplementary Material 8 [file 12967_2025_7507_MOESM8_ESM.pdf]

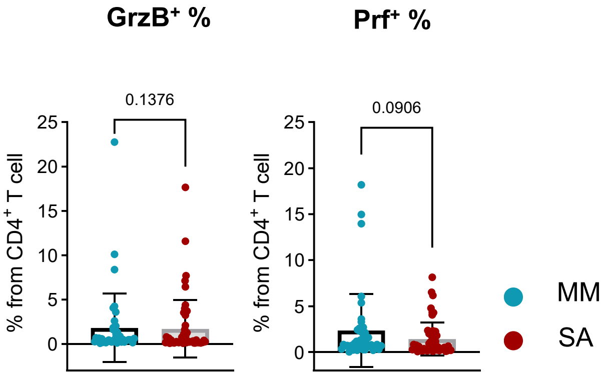

Supplementary Figure S6: Comparison of frequencies of cytotoxic mediators (granzyme B and perforin) in CD4⁺ T cells from people with mild/moderate (n=43) and severe ME/CFS (n=53). *Ex vivo* PBMC were stained with fluorescently labelled antibodies extracellularly for immune cell phenotyping, then stained with antibodies from the 'functional panel', comprised of granzyme B, perforin, IL-17 and Interferon-γ intracellularly. The frequencies of granzyme B and perforin were compared between the two groups in CD4⁺ T cells. Each dot represents the average value across all the samples collected at different time points for individual study participants. Mean values and SD are shown. Datasets were compared using the Mann-Whitney test for non-parametric data or the unpaired t-test for parametric data, with $p<0.05$ deemed significant. MM: people with mild/moderate symptoms; SA: severely affected people. GrzB; granzyme B, Prf; perforin.
