# Supplementary material for: Abnormal T-Cell activation and cytotoxic T-Cell frequency discriminate symptom severity in myalgic encephalomyelitis/chronic fatigue syndrome
Source: J Transl Med. 2025 Dec 10;24:68. doi: 10.1186/s12967-025-07507-x (PMC12801500; doi:10.1186/s12967-025-07507-x)
Supplement: Supplementary file 9 — Supplementary Material 9 [file 12967_2025_7507_MOESM9_ESM.pdf]

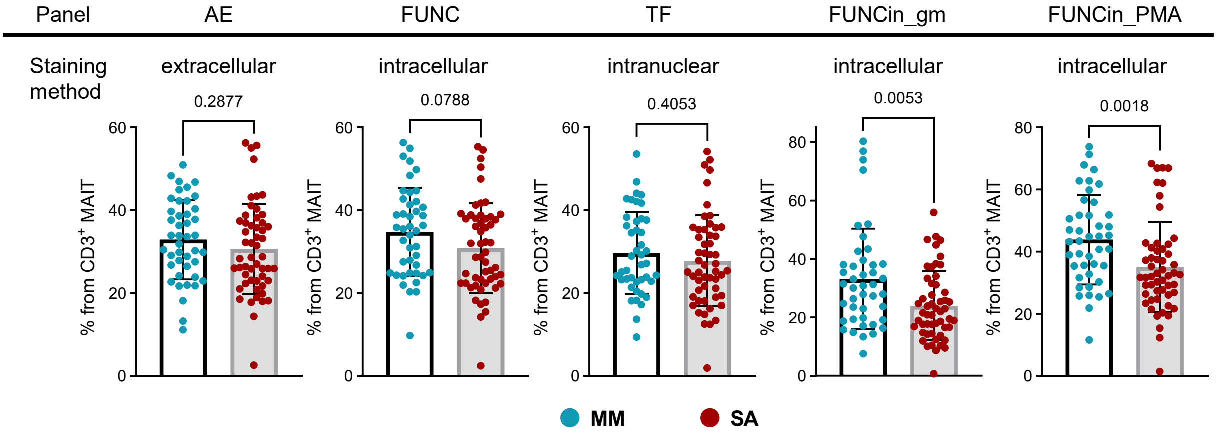

Supplementary Figure S7: Frequencies of CD4⁻CD8⁻ double negative (DN) mucosal-associated invariant T cell subsets in mild/moderate (n=43) and severe ME/CFS (n=53) in different staining methods. PBMC from people with mild/moderate (MM) or severe (SA) ME/CFS symptoms were analysed by flow cytometry, to determine the proportions of $CD4^-$ $CD8^-$ double negative T cells within the $CD3^+$ MAIT compartment. PBMC samples were analysed by four separate flow cytometry staining panels: in common, PBMCs were stained extracellularly for immune cell profiles, then specific stain methods were engaged. "AE": Activation/Exhaustion markers; "Func": functional markers; "TF": transcription factor markers; "FUNCin_gm": functional marker staining following incubation overnight in culture medium. "FUNCin_PMA": functional marker staining following 5-hour incubation with PMA+IM after incubation overnight. Each dot represents the average value across all the samples collected at different time points for one individual study participant. Mean values and SD were plotted in graphs. Datasets were compared using the Mann-Whitney test for non-parametric data or the unpaired t-test for parametric data. Statistical significance was set at $p < 0.05$. GM: Growth medium, PMA; phorbol 12-myristate 13-acetate, IM; ionomycin
