# Supplementary material for: Abnormal T-Cell activation and cytotoxic T-Cell frequency discriminate symptom severity in myalgic encephalomyelitis/chronic fatigue syndrome
Source: J Transl Med. 2025 Dec 10;24:68. doi: 10.1186/s12967-025-07507-x (PMC12801500; doi:10.1186/s12967-025-07507-x)
Supplement: Supplementary file 10 — Supplementary Material 10 [file 12967_2025_7507_MOESM10_ESM.pdf]

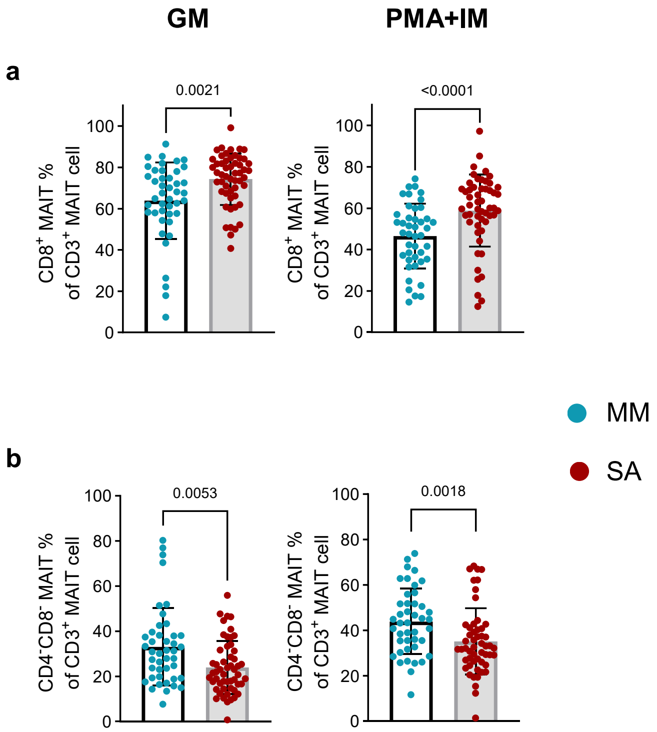

Supplementary Figure S8: Proportion changes of MAIT cell subsets after stimulation with PMA and ionomycin in people with mild/moderate (n=43) and severe ME/CFS (n=53). Thawed PBMCs were rested overnight and incubated with or without PMA and ionomycin for 5 hours. **(a)** Frequency of $CD8^{+}$MAIT cells **(b)** Frequency of $CD4^{-}CD8^{-}$MAIT cells. Each dot represents the average value across all the samples collected at different time points for individual study participants. Mean values and SD are shown. Datasets were compared using the Mann-Whitney test for non-parametric data or the t-test for parametric data, with $p<0.05$ deemed significant. MM: people with mild/moderate symptoms; SA: severely affected people. GM; growth medium, PMA; Phorbol 12-myristate 13-acetate, IM; ionomycin
