# Supplementary material for: Abnormal T-Cell activation and cytotoxic T-Cell frequency discriminate symptom severity in myalgic encephalomyelitis/chronic fatigue syndrome
Source: J Transl Med. 2025 Dec 10;24:68. doi: 10.1186/s12967-025-07507-x (PMC12801500; doi:10.1186/s12967-025-07507-x)
Supplement: Supplementary file 11 — Supplementary Material 11 [file 12967_2025_7507_MOESM11_ESM.pdf]

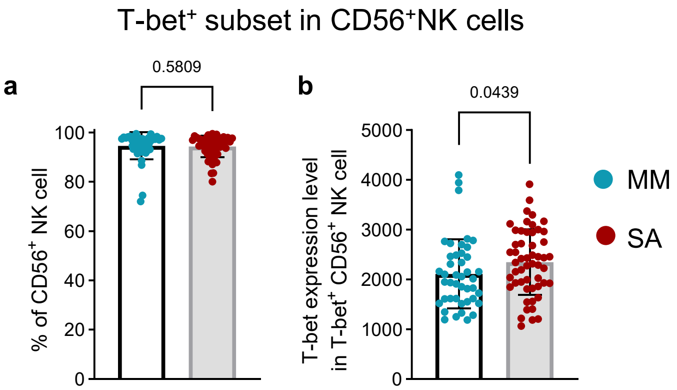

Supplementary Figure S9: Elevated median fluorescence of intensity (MFI) of T-bet expressing NK cells in people with severe ME/CFS. *Ex vivo* PBMC were stained for immune cell phenotyping, then intranuclearly stained with antibodies from the 'transcription factor panel'. The frequency of T-bet-expressing NK cells and MFI of T-bet were compared between the two clinical groups: **(a)** frequency of T-bet$^+$ population in NK cells; **(b)** MFI of T-bet expression in T-bet$^+$ NK cells. Each dot represents the average value across all the samples collected at different time points for individual study participants. Mean values and SD are shown. Datasets were compared using the Mann-Whitney test for non-parametric data, with $p<0.05$ deemed significant. MM: people with mild/moderate ME/CFS; SA: people severely affected by ME/CFS.
